# Association between *Helicobacter pylori* infection and rheumatoid arthritis: Results from the NHANES 1999–2000 and a Mendelian randomization study

**DOI:** 10.1097/MD.0000000000050458

**Published:** 2026-09-11

**Authors:** Jianhua Zhang, Weihua Li

**Affiliations:** a Department of Orthopedics, Nantong Second People’s Hospital, Nantong, China.

**Keywords:** causality, *Helicobacter pylori*, Mendelian randomization, NHANES, rheumatoid arthritis

## Abstract

The relationship between *Helicobacter pylori* infection and rheumatoid arthritis (RA) remains controversial. Our study aimed to provide evidence regarding the association between *H pylori* infection and RA by analyzing data from the 1999 to 2000 National Health and Nutrition Examination Survey (NHANES) and conducting Mendelian randomization (MR) analyses. A weighted logistic regression analysis was performed to examine the relationship between *H pylori* infection and RA, utilizing data from NHANES for the years 1999 to 2000. Furthermore, a bidirectional MR analysis was executed to explore both the causal and reverse causal relationships between *H pylori* infection and seropositive as well as seronegative RA. The primary analysis utilized inverse variance weighted in conjunction with additional MR methodologies to corroborate the causal link between *H pylori* infection and RA. Additionally, assessments of heterogeneity, pleiotropy, and sensitivity analyses were conducted to evaluate the robustness of the results. The cross-sectional observational analysis and MR study did not provide clear evidence to support a correlation and causal relationship between *H pylori* infection and RA. A total of 550 participants were enrolled in our observational study in NHANES. Following the adjustment for various confounding variables, the analysis revealed no statistically significant association between *H pylori* infection and RA (odds ratio = 1.300; 95% confidence interval = 0.784–2.154, *P* = .416) . In the MR analysis, 7 common serum *H pylori* antibodies (anti-*H pylori* immunoglobulin G, GroEL, OMP, UREA, CagA, VacA, and Catalase) were not found to have a causal relationship with either seropositive or seronegative RA. Moreover, reverse MR analysis did not suggest a causal effect of seropositive or seronegative RA on *H pylori* infection. Results were robust across all sensitivity analyses.

## 1. Introduction

Rheumatoid arthritis (RA) is a chronic autoimmune disorder that predominantly impacts the synovial joints. The principal clinical features of RA include synovitis, cartilage degradation, and joint destruction.^[1,2]^ The etiological factors associated with RA are multifaceted and may include infections, hormonal fluctuations, genetic susceptibilities, and environmental influences.^[3]^ Among these, the microbiota is recognized as a significant environmental factor. An expanding body of research indicates that the microbiome is instrumental in the initiation and progression of RA.^[4,5]^

*Helicobacter pylori*, a Gram-negative bacterium, represents one of the most common chronic bacterial infections in humans.^[6]^ The relationship between *H pylori* infection and the development of autoimmune disorders has garnered considerable attention in recent years. *H pylori* can induce persistent infection and chronic inflammation by stimulating immune responses.^[7]^

Research has indicated that patients diagnosed with RA who have successfully eradicated *H pylori* infection experience notable enhancements in joint symptoms, functional capacity, and various clinical metrics. Furthermore, laboratory markers have shown a progressive reduction. A significant association exists between *H pylori* infection and the pathogenesis of RA.^[8,9]^ In a study conducted by Ebrahimi et al, RA patients who tested positive for CagA related to *H pylori* exhibited more severe clinical symptoms, elevated DAS-28 values, and higher average VAS scores compared to their CagA-negative counterparts.^[10]^ Additionally, a 17-year retrospective cohort study revealed an increased risk of developing RA among individuals with *H pylori* infection when compared to a control group. This heightened prevalence of RA was particularly evident in patients under the age of 30, affecting both male and female populations.^[11]^

Numerous studies have investigated the association between *H pylori* infection and RA, yet the results remain ambiguous. Some research suggests a positive correlation between the 2 conditions, while other studies yield conflicting outcomes. For instance, a cohort study conducted in Japan indicated that individuals diagnosed with RA exhibited a lower prevalence of *H pylori* infection in comparison to healthy controls.^[12]^ Furthermore, a large cohort study encompassing 56,000 patients, both *H pylori* positive and negative, demonstrated comparable comorbidities across both groups. Notably, this study found no significant relationship between *H pylori* infection and RA, nor any differences in the prevalence of RA itself.^[13]^ Additionally, a meta-analysis conducted by Youssefi et al concluded that *H pylori* infection does not have a significant effect on the pathogenesis of RA, and no substantial correlation between *H pylori* infection and RA was established (odds ratio [OR] 1.18; 95% confidence interval [CI]: 0.91–1.52, *P*: 0.19).^[14]^

In order to explore the potential association between *H pylori* infection and RA, we first conducted a cross-sectional study utilizing data from National Health and Nutrition Examination Survey (NHANES) to identify any observational correlations between *H pylori* infection and RA. Following this, we employed Mendelian randomization (MR) to examine the causal relationship between 7 distinct classes of *H pylori* antibodies and RA. MR is a methodological approach within epidemiology that facilitates causal inference by using genetic variants as instrumental variables (IVs). This technique aims to elucidate causal relationships between exposures and outcomes, reduce the impact of confounding variables, and enhance the strength of the associations observed between the exposure and the outcome.^[15]^

## 2. Methods

### 2.1. Study population in NHANES

**Figure 1.**
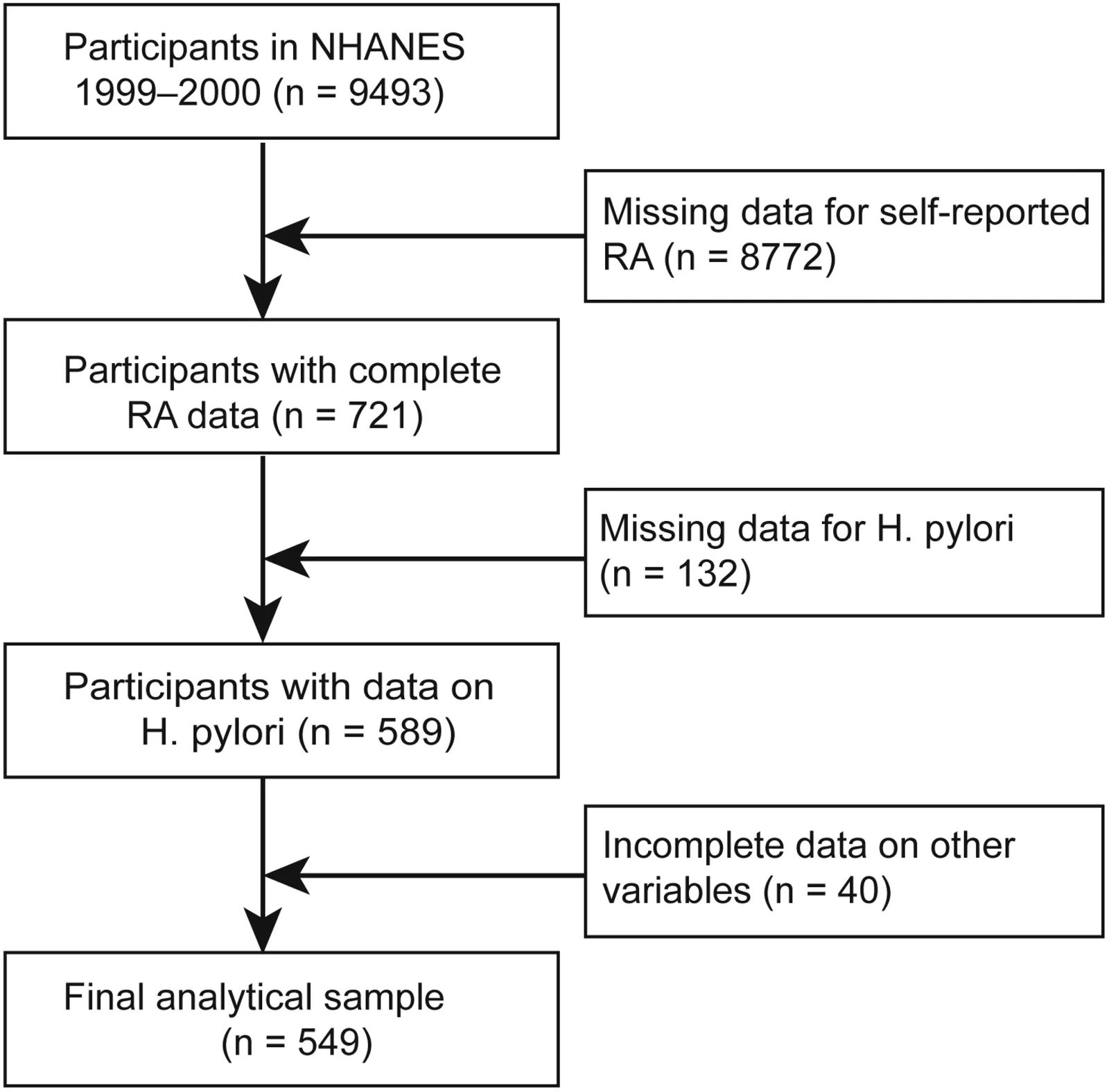
Inclusion and exclusion flow chart of NHANES participants. NHANES = National Health and Nutrition Examination Survey, RA = rheumatoid arthritis.

The NHANES database is a cross-sectional survey of the United States population, designed to assess health and nutritional status. In the present study, data were collected from 1999 to 2000 and included a total of 9493 individuals. After the exclusion of records lacking an RA diagnosis and an identification of a history of *H pylori* infection, a total of 589 samples were obtained. Subsequently, an additional 39 individuals with missing covariate information (age, race, sex, body mass index [BMI], smoking, drinking, hypertension, diabetes) were excluded, resulting in a final sample size of 550 individuals (Fig. 1). Further details may be found at https://wwwn.cdc.gov/nchs/nhanes/Default.aspx.

### 2.2. Definition and assessment of RA and *H pylori* infection in NHANES

The diagnosis of RA was based on the MCQ190 questionnaire. If a participant responded “yes” to the question “Doctor ever said you had arthritis?” with “yes,” and the question “Which type of arthritis?” or “Which type of arthritis was it?” with “RA,” they were diagnosed as having RA. The diagnosis of *H pylori* infection was based on the quantification of immunoglobulin (Ig)G antibodies to *H pylori* in the serum of 7439 participants. The optical density values obtained from the ELISA analysis of all participants ranged from 0 to 5.73. Values exceeding 1.1 were deemed indicative of seropositive, and values < 0.9 were deemed indicative of seronegative. Conversely, values between 0.9 and 1.1 were classified as equivocal. To prevent misleading statistical outcomes, participants with equivocal optical density values (N = 170) were excluded from the study.

### 2.3. Other covariates used in NHANES

The covariates included in our study that may influence RA include age, gender (male, female), race (Mexican American, non-Hispanic White, non-Hispanic Black, other Hispanic, and other race), BMI smoking (yes, no), alcohol (yes, no), hypertension (yes, no), and diabetes (yes, no).

### 2.4. Exposure and outcome genome-wide association study (GWAS) data source in MR

**Figure 2.**
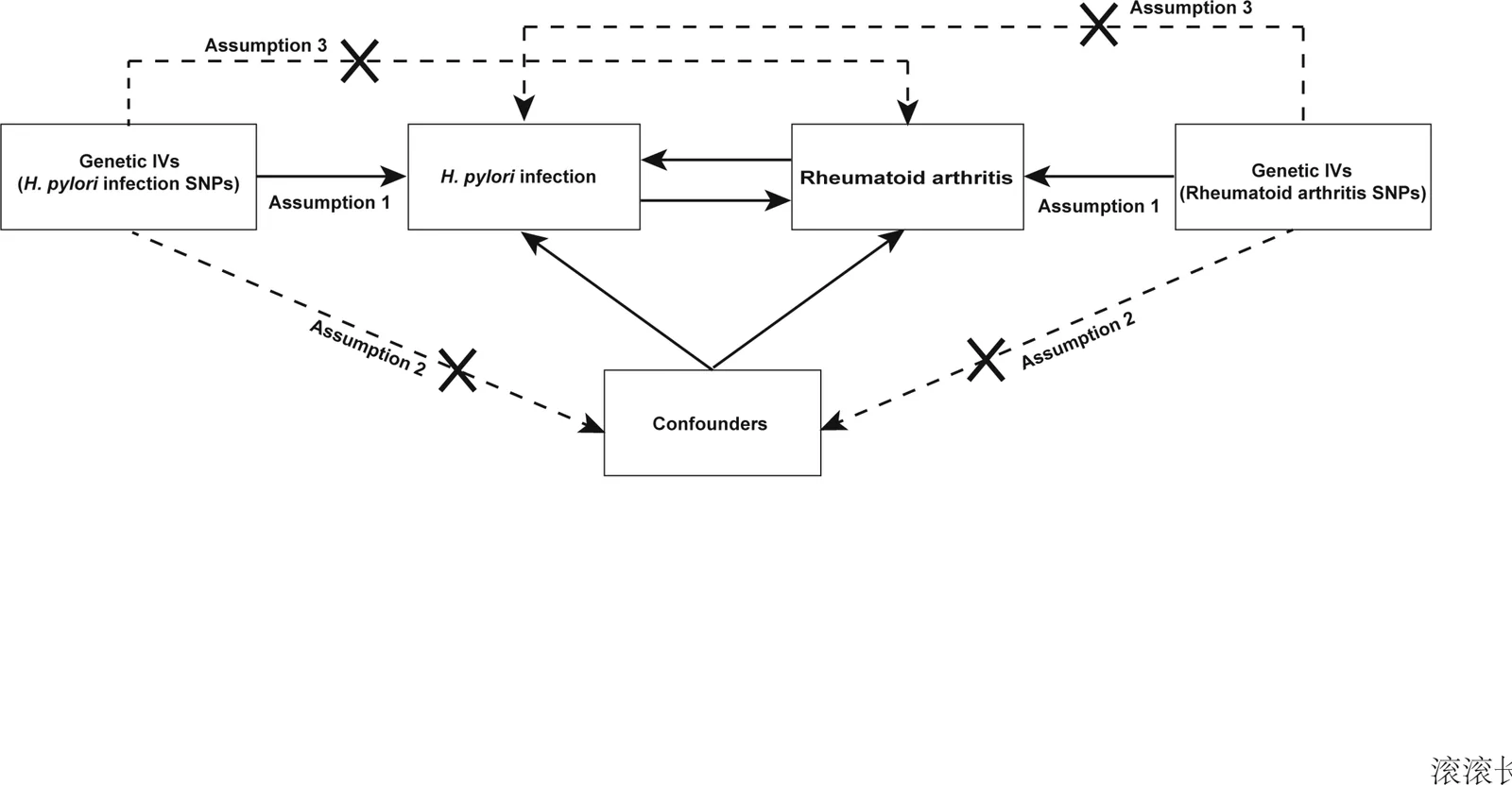
Schematic representation of the bidirectional MR study on the causal relationship between *Helicobacter pylori* infection and RA. Assumption 1: IVs are associated with the exposure. Assumption 2: IVs are not associated with confounders. Assumption 3: IVs affect the outcome only through the exposure. IV = instrument variable, MR = Mendelian randomization, RA = rheumatoid arthritis, SNP = single nucleotide polymorphism.

The present study is a bidirectional MR study, and MR must fulfill 3 major assumptions: the assumption of correlation (the selected IVs are strongly correlated with exposure, with *P* < 1e−6 and *F* > 10); the assumption of independence (there is no correlation between the IVs and the outcome); and the assumption of exclusivity (the IVs cannot influence the outcome through confounders other than exposure) (Fig. 2). Genetic variants associated with seropositive and seronegative RA were extracted from GWAS studies, including seropositive (18,019 cases and 991,604 controls) and seronegative (8515 cases and 1,015,471 controls). All participants were of European ancestry. Instrumental single nucleotide polymorphisms (SNPs) were associated with RA at genome-wide significance (*P* < 1e−6). We used the summary GWAS data for 7 antibodies against *H pylori* (anti-*H pylori* IgG, GroEL, OMP, UreA, CagA, VacA, and Catalase) presented in Butler-Laporte et al, comprehensively covering multiple phenotypes of *H pylori* infection^[17]^. All these summary data can be found and downloaded from the IEU OpenGWAS project (https://gwas.mrcieu.ac.uk/). Details of the included GWAS data in this study are provided in Supplementary Table S1, Supplemental Digital Content 1.

### 2.5. Selection of IVs in MR

A series of quality control measures were implemented to select eligible IVs for RA and *H pylori* infection that meet the assumptions of MR analysis. SNPs with genome-wide association (*P* < 1e−6) were extracted, and SNPs with weak IVs for exposure were excluded. We did not select a genome-wide significance threshold (*P* < 5 × 10^−8^) due to insufficient SNPs available for MR analysis. Then, we clumped the GWAS significant SNPs from each split’s GWAS with a clumping window of 10,000 kb and an *R*^2^ threshold of 0.001. Furthermore, in order to prevent any potential bias arising from weak instruments, the *F*-statistics of the SNPs were computed using the following formula: *FF* = [(n − k − 1)/ k] * [*R*^2^/ 1 − *R*^2^]. The *F*-statistic values are indicative of the strength of the IVs; values < 10 are typically considered to indicate weak IVs and thus are excluded. The SNP proxy was not utilized in this analysis. (Supplementary Table S3–S4, Supplemental Digital Content 2).

### 2.6. Confounder removal

Prior to conducting the MR analysis, any SNPs that were found to be associated with confounders (BMI, cardiovascular diseases, diabetes, dyslipidemia, drinking, and smoking) were removed in order to minimize the potential for such factors to interfere with the results. The GWAS Catalog database was used to exclude confounding factors because the Phenoscanner dataset was not accessible for unknown reasons (https://www.ebi.ac.uk/gwas/). Finally, the exposed SNPS work synergistically with the resulting SNPS to eliminate palindromic sequences and incompatible SNPS.

## 3. Statistical analysis

In observational epidemiological analyses, the baseline characteristics of all study participants were described using either a mean value (for continuous variables) or a proportion (for categorical variables). A logistic regression model was employed to calculate the OR, *P* values, and 95% CIs between *H pylori* infection and RA risk. Given the complex probabilistic clustering design of NHANES, weights (WTMEC2YR) were considered in statistical analyses in this study.

With *H pylori* infection-associated IVs as exposure and RA as outcome, forward MR analysis was performed to detect the causal relationship between *H pylori* infection and RA at the genetic level. The SNPs closely related to RA were used as IVs, and the 7 phenotypes associated with *H pylori* infection were used as results for reverse MR Analysis. To examine a potential causal association, the inverse variance weighted (IVW), MR-Egger, weighted median, weighted mode, and simple mode were employed. The IVW was identified as the primary analytical method. Subsequently, MR-Egger regression was utilized to assess potential directional pleiotropy by examining the intercept term.^[16]^

MR-pleiotropy residual sum and outlier (MR-PRESSO) can be used to identify and remove possible pleiotropic SNPs detected as outliers in MR analysis.^[17]^ The heterogeneity among the IV was evaluated using the Cochran *Q* test and the *I*^2^ test.^[18]^ Furthermore, a sensitivity analysis was conducted using the leave-one-out method to assess the influence of each SNP. In the statistical inference, Bonferroni or FDR multiple-testing correction was not employed.

All statistical analyses were performed using *R* software (version 4.4.1). The core packages employed were TwoSampleMR and Survey. A significance level of *P* < .05 was considered indicative of a statistically significant result. All the data for this study were publicly available summary statistics; therefore, ethical approval and consent to participate were not required.

## 4. Results

### 4.1. Epidemiological observation and analysis

Comprehensive information pertaining to the inclusion and exclusion criteria for study subjects is delineated in Figure 1. As a result, a total of 550 participants were ultimately incorporated into this cross-sectional study. The specific characteristics of the participants included in the study are presented in Supplementary Table S2, Supplemental Digital Content 3. Furthermore, Table 1 depicts the association between *H pylori* infection and RA. In Model 1 (OR = 1.268, *P* = .427), Model 2 (OR = 1.276, *P* = .429) and Model 3 (OR = 1.300, *P* = .416). The findings from the weighted logistic regression analysis revealed no statistically significant correlation between *H pylori* infection and the incidence of RA.

**Table 1:** Association between *Helicobacter pylori* infection and RA.

*H pylori*	Model 1: OR (95% CI)	*P* value	Model 2: OR (95% CI)	*P* value	Model 3: OR (95% CI)	*P* value
Negative	Reference		Reference		Reference	
Positive	1.268 (0.719–2.237)	.427	1.276 (0.722–2.256)	.429	1.300 (0.784–2.154)	.416

Model 1 adjusted for: none. Model 2 adjusted for: gender, age, race. Model 3 adjusted for: gender, age, race, BMI, smoking, alcohol, hypertension, diabetes.

BMI = body mass index, CI = confidence interval, OR = odds ratio, RA = rheumatoid arthritis.

### 4.2. The causal effect of *H pylori* infection on seropositive RA risk

Utilizing a rigorous significance threshold of *P* < 1 × 10 − 6, the analysis identified 8, 6, 10, 8, 10, 4, and 14 IVs corresponding to the antibody levels of anti-*H pylori* CagA, OMP, UREA, Catalase, IgG, GroEL, and VacA, respectively. The *F*-statistics for all SNPs exceeded 10, suggesting the absence of weak IVs (Supplementary Table S3, Supplemental Digital Content 2). MR analysis conducted for each of these 7 phenotypes associated with *H pylori* infection as exposure factors did not provide evidence supporting a causal relationship between *H pylori* infection and seropositive RA. The results of the IVW method showed the following ORs and CIs for *H pylori* CagA antibody (OR = 0.984, *P* = .698), *H pylori* OMP antibody (OR = 0.909, *P* = .264), *H pylori* UREA antibody (OR = 0.958, *P* = .384), *H pylori* Catalase antibody (OR = 0.953, *P* = .259), Anti-*H pylori* IgG (OR = 1.003, *P* = .989), *H pylori* GroEL antibody (OR = 0.907, *P* = .401), and *H pylori* VacA antibody (OR = 0.984, *P* = .142) (Table 2). Comparable findings were noted in the MR-Egger regression, weighted median, weighted mode, and simple mode analyses, as detailed in Table 2. There was no indication of horizontal pleiotropy in the MR-Egger regression, nor was it identified in the MR-PRESSO analysis (*P* values: .245, .214, .587, .657, .760, .206, .229). The *P* values for both MR-Egger and IVW exceeded .05, suggesting an absence of significant heterogeneity among the IVs. A scatter plot and funnel plot depicting these results are provided in Supplementary Figure S1–S2, Supplemental Digital Content 4. Furthermore, the leave-one-out sensitivity analysis demonstrated that the overall results remained consistent regardless of the exclusion of any individual SNP, thereby reinforcing the credibility of the findings (Supplementary Fig. S3, Supplemental Digital Content 5).

**Table 2:** Main MR results when *Helicobacter pylori* is considered as exposure.

Exposure	Outcome	Methods	OR (95% CI)	*P* value	*P* value for heterogeneity test	*P* value for pleiotropy test	*P* value for MR-PRESSO
CagA	Seropositive RA	MR-Egger	0.981 (0.817–1.177)	.841	.134	.971	.245
		Weighted median	1.001 (0.909–1.102)	.981			
		IVW	0.984 (0.905–1.069)	.698	.201		
		Simple mode	0.978 (0.843–1.133)	.772			
		Weighted mode	1.034 (0.919–1.164)	.596			
OMP		MR Egger	0.752 (0.499–1.132)	.244	.717	.377	.214
		Weighted median	0.884 (0.711–1.100)	.269			
		IVW	0.909 (0.768–1.075)	.264	.686		
		Simple mode	0.822 (0.611–1.105)	.250			
		Weighted mode	0.898 (0.654–1.233)	.536			
							
UREA		MR Egger	0.949 (0.725–1.242)	.711	.469	.941	.587
		Weighted median	0.988 (0.872–1.120)	.851			
		IVW	0.958 (0.870–1.055)	.384	.570		
		Simple mode	0.998 (0.820–1.215)	.987			
		Weighted mode	1.004 (0.844–1.193)	.968			
							
Catalase		MR Egger	0.903 (0.775–1.053)	.242	.635	.446	.657
		Weighted median	0.932 (0.842–1.032)	.174			
		IVW	0.953 (0.877–1.036)	.259	.663		
		Simple mode	0.947 (0.823–1.091)	.476			
		Weighted mode	0.918 (0.815–1.035)	.205			
IgG		MR Egger	0.403 (0.095–1.707)	.252	.805	.233	.760
		Weighted median	0.850 (0.507–1.426)	.538			
		IVW	1.003 (0.669–1.503)	.989	.719		
		Simple mode	0.882 (0.392–1.987)	.770			
		Weighted mode	0.852 (0.402–1.807)	.686			
							
GroEL		MR Egger	1.261 (0.755–2.104)	.469	.211	.305	.206
		Weighted median	0.996 (0.823–1.207)	.971			
		IVW	0.907 (0.723–1.138)	.401	.111		
		Simple mode	1.060 (0.770–1.460)	.744			
		Weighted mode	1.060 (0.790–1.423)	.723			
VacA		MR Egger	0.879 (0.767–1.008)	.090	.313	.233	.229
		Weighted median	0.977 (0.883–1.080)	.648			
		IVW	0.948 (0.882–1.018)	.142	.270		
		Simple mode	1.010 (0.843–1.210)	.913			
		Weighted mode	1.003 (0.840–1.198)	.973			
CagA	Seronegative RA	MR Egger	1.238 (0.939–1.631)	.168	.059	.260	.266
		Weighted median	1.033 (0.889–1.199)	.675			
		IVW	1.065 (0.932–1.216)	.354	.038		
		Simple mode	0.927 (0.710–1.211)	.593			
		Weighted mode	0.942 (0.764–1.161)	.587			
OMP		MR Egger	1.157 (0.634–2.111)	.660	.544	.477	.710
		Weighted median	0.825 (0.605–1.124)	.223			
		IVW	0.929 (0.726–1.189)	.559	.594		
		Simple mode	0.809 (0.531–1.232)	.368			
		Weighted mode	0.813 (0.560–1.180)	.326			
UREA		MR Egger	0.875 (0.589–1.299)	.526	.850	.320	.834
		Weighted median	1.094 (0.904–1.324)	.355			
		IVW	1.068 (0.927–1.231)	.363	.816		
		Simple mode	1.229 (0.896–1.686)	.233			
		Weighted mode	1.222 (0.911–1.638)	.214			
Catalase		MR Egger	1.148 (0.914–1.441)	.280	.456	.119	.310
		Weighted median	1.002 (0.851–1.179)	.983			
		IVW	0.961 (0.835–1.106)	.580	.251		
		Simple mode	0.986 (0.783–1.242)	.908			
		Weighted mode	1.020 (0.844–1.233)	.841			
IgG		MR Egger	0.222 (0.026–1.900)	.212	.431	.388	.488
		Weighted median	0.605 (0.262–1.393)	.237			
		IVW	0.583 (0.313–1.087)	.090	.451		
		Simple mode	0.417 (0.124–1.399)	.194			
		Weighted mode	0.539 (0.159–1.828)	.350			
GroEL		MR Egger	0.823 (0.448–1.547)	.620	.507	.963	.733
		Weighted median	0.862 (0.654–1.136)	.291			
		IVW	0.845 (0.666–1.072)	.165	.715		
		Simple mode	0.888 (0.591–1.334)	.607			
		Weighted mode	0.892 (0.613–1.297)	.591			
VacA		MR Egger	0.948 (0.787–1.143)	.588	.881	.493	.886
		Weighted median	1.071 (0.937–1.224)	.318			
		IVW	1.005 (0.913–1.106)	.921	.895		
		Simple mode	1.101 (0.876–1.384)	.423			
		Weighted mode	1.100 (0.889–1.360)	.396			

CI = confidence interval, Ig = immunoglobulin, IVW = inverse variance weighted, MR = Mendelian randomization, MR-PRESSO = MR-pleiotropy sum and outlier, OR = odds ratio, RA = rheumatoid arthritis.

### 4.3. The causal effect of *H pylori* infection on Seronegative RA risk

The findings suggest that *H pylori* infection is not causally related to seronegative RA. The *F*-statistics of all eligible SNPs and SNPs are shown in Supplementary Table S3, Supplemental Digital Content 2. IVW results were as follows: *H pylori* CagA antibody (OR = 1.065, *P* = .354), *H pylori* OMP antibody (OR = 0.929, *P* = .559), *H pylori* UREA antibody (OR = 1.068, *P* = .363), *H pylori* Catalase antibody (OR = 0.961, *P* = .580), Anti-*H pylori* IgG (OR = 0.583, *P* = .090), *H pylori* GroEL antibody (OR = 0.845, *P* = .165), and *H pylori* VacA antibody (OR = 1.005, *P* = .921) (Table 2). The MR-PRESSO and MR-Egger intercept tests revealed no indications of potential horizontal pleiotropy, as all *P* values (.266, .710, .834, .310, .488, .733, 0.886) were not significant (Table 2). Although nominal heterogeneity was observed in the IVW method of *H pylori* CagA antibody in seronegative RA (*P* = .038), the results remained robust in the random-effects IVW model and subsequent sensitivity analyses (leave-one-out and MR-PRESSO), supporting the null association (Table 2). The overall results were stable even after the exclusion of individual SNPs through the leave-one-out method (Supplementary Fig. S4–S6, Supplemental Digital Content 6).

### 4.4. The causal effect of RA on *H pylori* infection risk

As illustrated in Table 3, the reverse MR analysis, which considered seropositive RA and seronegative RA as exposure variables and *H pylori* as the outcome, consistently yielded negative results. This indicates a lack of evidence supporting a causal relationship between RA and *H pylori* infection. The findings from the Cochran *Q* test demonstrated no significant heterogeneity among the studies. Additionally, MR-Egger regression did not provide any evidence of pleiotropy. The MR-PRESSO analysis was found to be robust, with no outliers identified. The *F*-statistics for all eligible SNPs, leave-one-out analyses, along with forest and funnel plots are detailed in Supplementary Table S4, Supplemental Digital Content 7, Figure S7–S12, Supplemental Digital Content 8.

**Table 3:** Main MR results when RA is considered as exposure.

Exposure	Outcome	Methods	OR (95% CI)	*P* value	*P* value for heterogeneity test	*P* value for pleiotropy test	*P* value for MR-PRESSO
Seropositive RA	CagA	MR Egger	1.026 (0.921–1.142)	.646	.719	.653	.738
		Weighted median	0.971 (0.884–1.067)	.540			
		IVW	1.007 (0.939–1.079)	.855	.756		
		Simple mode	1.148 (0.918–1.436)	.236			
		Weighted mode	0.997 (0.901–1.105)	.960			
	OMP	MR Egger	1.041 (0.973–1.115)	.254	.439	.477	.595
		Weighted median	1.026 (0.968–1.087)	.386			
		IVW	1.022 (0.978–1.067)	.337	.466		
		Simple mode	1.026 (0.909–1.159)	.682			
		Weighted mode	1.032 (0.981–1.086)	.233			
	UREA	MR Egger	0.974 (0.903–1.051)	.507	.336	.952	.346
		Weighted median	0.958 (0.897–1.023)	.203			
		IVW	0.976 (0.930–1.024)	.324	.389		
		Simple mode	0.992 (0.861–1.143)	.912			
		Weighted mode	0.951 (0.891–1.014)	.139			
	Catalase	MR Egger	1.052 (0.965–1.146)	.263	.829	.113	.746
		Weighted median	1.032 (0.958–1.111)	.411			
		IVW	0.996 (0.941–1.053)	.877	.741		
		Simple mode	1.097 (0.931–1.292)	.279			
		Weighted mode	1.025 (0.958–1.098)	.478			
							
	IgG	MR Egger	0.996 (0.977–1.015)	.690	.118	.925	.203
		Weighted median	0.995 (0.980–1.009)	.458			
		IVW	0.997 (0.985–1.009)	.603	.146		
		Simple mode	1.002 (0.972–1.032)	.920			
		Weighted mode	0.998 (0.984–1.012)	.757			
	GroEL	MR Egger	0.978 (0.916–1.044)	.506	.969	.714	.989
		Weighted median	0.967 (0.915–1.021)	.227			
		IVW	0.968 (0.928–1.011)	.141	.977		
		Simple mode	1.004 (0.912–1.105)	.934			
		Weighted mode	0.972 (0.920–1.026)	.314			
	VacA	MR Egger	1.046 (0.957-1.143)	.331	.453	.239	
		Weighted median	1.002 (0.928-1.082)	.956			
		IVW	1.003 (0.947-1.063)	.914	.429		.928
		Simple mode	1.088 (0.939-1.261)	.271			
		Weighted mode	1.004 (0.937-1.077)	.903			
Seronegative RA	CagA	MR Egger	0.962 (0.841–1.101)	.581	.423	.417	.216
		Weighted median	0.949 (0.836–1.076)	.412			
		IVW	1.006 (0.927–1.093)	.883	.443		
		Simple mode	0.946 (0.760–1.177)	.622			
		Weighted mode	0.955 (0.835–1.092)	.511			
	OMP	MR Egger	1.079 (0.984–1.182)	.122	.381	.052	.231
		Weighted median	1.041 (0.960–1.129)	.327			
		IVW	1.001 (0.940–1.065)	.984	.205		
		Simple mode	0.991 (0.853–1.151)	.907			
		Weighted mode	1.051 (0.963–1.147)	.278			
	UREA	MR Egger	0.950 (0.855–1.054)	.345	.186	.182	.159
		Weighted median	0.997 (0.911–1.091)	.945			
		IVW	1.007 (0.944–1.075)	.830	.144		
		Simple mode	1.018 (0.883–1.173)	.812			
		Weighted mode	1.003 (0.917–1.097)	.953			
	Catalase	MR Egger	1.039 (0.917–1.176)	.555	.333	.761	.364
		Weighted median	0.976 (0.884–1.078)	.635			
		IVW	1.023 (0.952–1.098)	.541	.387		
		Simple mode	0.922 (0.777–1.095)	.365			
		Weighted mode	0.961 (0.847–1.089)	.540			
	IgG	MR Egger	0.990 (0.967–1.014)	.413	.165	.107	.119
		Weighted median	0.996 (0.977–1.015)	.664			
		IVW	1.006 (0.991–1.021)	.442	.096		
		Simple mode	0.991 (0.963–1.020)	.539			
		Weighted mode	0.996 (0.977–1.015)	.676			
	GroEL	MR Egger	0.971 (0.894–1.054)	.490	.419	.490	.457
		Weighted median	0.956 (0.889–1.028)	.224			
		IVW	0.994 (0.945–1.045)	.805	.451		
		Simple mode	0.989 (0.868–1.127)	.866			
		Weighted mode	0.959 (0.891–1.032)	.273			
	VacA	MR Egger	1.041 (0.941–1.153)	.444	.883	.667	
		Weighted median	1.028 (0.935–1.130)	.567			
		IVW	1.023 (0.959–1.091)	.484	.908		
		Simple mode	1.068 (0.918–1.244)	.405			
		Weighted mode	1.023 (0.927–1.130)	.652			

CI = confidence interval, Ig = immunoglobulin, IVW = inverse variance weighted, MR = Mendelian randomization, MR-PRESSO = MR-pleiotropy sum and outlier, OR = odds ratio, RA = rheumatoid arthritis.

## 5. Discussions

To the best of our understanding, this research represents the inaugural effort to combine stepwise analyses of the NHANES study with a bidirectional MR study to examine the causal relationship between *H pylori* infection and RA. The results of our investigation did not provide evidence for a causal link between *H pylori* infection and the risk of developing RA. Multiple MR sensitivity analysis methods showed consistent results.

It is well established that infections can serve as triggers or aggravating factors for autoimmune diseases. Chronic infection with *H pylori* may play a significant role in the pathogenesis of certain autoimmune disorders and in the maintenance of inflammatory activity.^[7]^ Yamanishi et al presented various B-1 cell-associated autoreactive antibodies, such as IgM-type rheumatoid factor, when splenic B cells were stimulated with purified *H pylori* urease in vitro.^[19]^ Wu et al first showed that *H pylori* infection induces citrullination of cellular proteins by upregulating the protein arginine deiminase 4 (PAD4). PAD4 interacts with *H pylori* to up-regulate PAD4 expression by stabilizing HIF-1α to aggravate RA.^[20]^ Seriolo et al found that RA patients who eradicated *H pylori* infection had significantly improved clinical and laboratory test results compared with *H pylori* negative patients.^[21]^

While other studies have shown a different trend, the presence of a rheumatoid factor was inversely related to *H pylori* infection and the value of the rheumatoid factor.^[22]^ Similarly, a systematic review and meta-analysis study did not find a significant correlation between *H pylori* infection and RA. *H pylori* infection has no significant effect on the development of RA, especially in countries with high *H pylori* colonization rates, where the prevalence of RA is low.^[14]^ In a 62-year-old Japanese woman with RA who received eradication therapy with *H pylori*, measures of RA activity worsened: significant increases in C-reactive protein, RA precipitation antigen, erythrocyte sedimentation rate, and complement CH50.^[23]^ Razavi et al reported that after successful eradication of *H pylori*, the patient developed reactive arthritis, probably due to an opportunistic infection, possibly due to Clostridium difficile.^[24]^

In summary, the association between *H pylori* infection and RA continues to be a subject of debate. This debate is largely attributed to the vulnerability of both prospective and cross-sectional studies to confounding variables, including environmental factors and selection bias, which complicate the determination of a clear causal relationship. Furthermore, the diagnostic criteria employed for *H pylori* infection were inconsistent across the various studies.

The main strengths of this study include the large cross-sectional study based on NHANES and the MR analysis study. Second, summary statistics of 7 phenotypes associated with *H pylori* infection were used to expand the *H pylori* infection list, and statistical power was improved by using the most recent and complete GWAS data as IVs. However, this study has some unavoidable limitations. Several limitations of this study should be noted. First, the analytical sample size from the NHANES database was relatively small, and the diagnosis of RA relied on self-reported data, which may introduce recall or misclassification bias. In contrast, the subsequent bidirectional MR analyses, which rely on genetic IV largely free from recall bias and misclassification, provide more robust evidence for causal inference. But, the current sample size and number of instruments limit the ability to detect small effects. Second, *H pylori* exposure was based on serology testing, which reflects historical exposure rather than active infection status. Third, in the MR analysis, we adopted a relaxed significance threshold for secondary instrument selection (*P* < 1e−6) to ensure sufficient statistical power, which might introduce suggestive or weaker IV. Finally, we did not apply strict multiple-testing corrections across all analyses (Bonferroni or FDR correction). While these limitations do not invalidate our robust null findings, they necessitate caution when generalizing the results, and further large-scale prospective studies with clinical gold-standards are warranted.

## 6. Conclusions

The results of our study indicated that no clear evidence of a causal association was detected between genetically predicted *H pylori* infection and RA.

## Acknowledgments

We would like to thank the participants and investigators of the database we used in this study.

## Author contributions

**Conceptualization:** Jianhua Zhang, Weihua Li.

**Data curation:** Jianhua Zhang, Weihua Li.

**Formal analysis:** Jianhua Zhang, Weihua Li.

**Funding acquisition:** Jianhua Zhang, Weihua Li.

**Investigation:** Jianhua Zhang, Weihua Li.

**Methodology:** Jianhua Zhang, Weihua Li.

**Project administration:** Jianhua Zhang, Weihua Li.

**Resources:** Jianhua Zhang, Weihua Li.

**Software:** Jianhua Zhang, Weihua Li.

**Supervision:** Jianhua Zhang, Weihua Li.

**Validation:** Jianhua Zhang, Weihua Li.

**Visualization:** Jianhua Zhang, Weihua Li.

**Writing – original draft:** Jianhua Zhang, Weihua Li.

**Writing – review & editing:** Jianhua Zhang, Weihua Li.
































