# Supplementary material for: Association between Helicobacter pylori infection and rheumatoid arthritis: Results from the NHANES 1999–2000 and a Mendelian randomization study
Source: Medicine (Baltimore). 2026 Sep 11;105(37):e50458. doi: 10.1097/MD.0000000000050458 (PMC13574391; doi:10.1097/MD.0000000000050458)
Supplement: Supplementary file 1 [file medi-105-e50458-s001.docx]

| **Supplementary Table S1** Characteristics of GWAS enrolled in the MR study | | | | | |
| --- | --- | --- | --- | --- | --- |
| **Items** | **GWAS ID** | **Year** | **Sample size** | **SNPs** | **Population** |
| Seropositive rheumatoid arthritis | finn-b-RHEUMA_SEROPOS | 2021 | 177,430 | 16,380,319 | European |
| Seronegative rheumatoid arthritis | finn-b-RHEUMA_SERONEG | 2021 | 174,771 | 16,380,301 | European |
| Anti-helicobacter pylori IgG seropositivity | ebi-a-GCST90006910 | 2020 | 8,735 | 9,170,312 | European |
| Helicobacter pylori CagA antibody levels | ebi-a-GCST90006911 | 2020 | 985 | 9,165,056 | European |
| Helicobacter pylori Catalase antibody levels | ebi-a-GCST90006912 | 2020 | 1,558 | 9,167,570 | European |
| Helicobacter pylori GroEL antibody levels | ebi-a-GCST90006913 | 2020 | 2,716 | 9,172,299 | European |
| Helicobacter pylori OMP antibody levels | ebi-a-GCST90006914 | 2020 | 2,640 | 9,167,440 | European |
| Helicobacter pylori UREA antibody levels | ebi-a-GCST90006915 | 2020 | 2,251 | 9,170,248 | European |
| Helicobacter pylori VacA antibody levels | ebi-a-GCST90006916 | 2020 | 1,571 | 9,178,635 | European |
| GWAS: Genome-wide association study; SNPs: Single nucleotide polymorphisms. | | | | | |
