# Supplementary material for: Association between Helicobacter pylori infection and rheumatoid arthritis: Results from the NHANES 1999–2000 and a Mendelian randomization study
Source: Medicine (Baltimore). 2026 Sep 11;105(37):e50458. doi: 10.1097/MD.0000000000050458 (PMC13574391; doi:10.1097/MD.0000000000050458)
Supplement: Supplementary file 2 [file medi-105-e50458-s002.docx]

| Supplementary Table S3 Summary statistics of genetic instruments when antibodies against H. pylori infection are considered as exposure | | | | | | | | | | | | | | | | | |
| --- | --- | --- | --- | --- | --- | --- | --- | --- | --- | --- | --- | --- | --- | --- | --- | --- | --- |
| **Exposure** | **Instrumental variables of Exposure** | | | **Allele effects on Exposure** | | | | | | | **Allele effects on Outcome** | | | | | | |
|  | **rsid** | **Chromosome** | **Position** | **effect_allele.exposure** | **other_allele.exposure** | **eaf.exposure** | **beta.exposure** | **se.exposure** | **pval.exposure** | ***F*** | outcome | **effect_allele.outcome** | **other_allele.outcome** | **eaf.outcome** | **beta.outcome** | **se.outcome** | **pval.outcome** |
| CagA | rs117827497 | 11 | 105212623 | G | A | 0.030462 | -0.583731 | 0.125147 | 3.09585E-06 | 21.7121043 | Seropositive RA | G | A | 0.01454 | 0.0281 | 0.0913 | 0.758 |
|  | rs118006294 | 16 | 10974221 | C | T | 0.050367 | -0.475295 | 0.101815 | 3.03837E-06 | 21.74804622 |  | C | T | 0.02991 | 0.0194 | 0.0675 | 0.7734 |
|  | rs11858369 | 15 | 90495509 | G | A | 0.068381 | 0.437721 | 0.0864978 | 4.18177E-07 | 25.55653255 |  | G | A | 0.08047 | 0.0509 | 0.0423 | 0.229 |
|  | rs149747348 | 12 | 98890443 | C | G | 0.013186 | -0.895407 | 0.194003 | 3.92292E-06 | 21.25892825 |  | C | G | 0.04026 | -0.0368 | 0.0569 | 0.5181 |
|  | rs4268452 | 10 | 81319354 | T | C | 0.071203 | -0.384976 | 0.0840329 | 4.62189E-06 | 20.94526676 |  | T | C | 0.06257 | 0.1123 | 0.0462 | 0.0150501 |
|  | rs6530847 | 8 | 15111565 | A | T | 0.391211 | -0.210266 | 0.0452734 | 3.41154E-06 | 21.52628924 |  | A | T | 0.2757 | -0.0156 | 0.0251 | 0.535499 |
|  | rs75170215 | 3 | 25908239 | C | T | 0.02057 | 0.743256 | 0.156416 | 2.01614E-06 | 22.53366242 |  | C | T | 0.02846 | -0.0854 | 0.0686 | 0.2135 |
|  | rs75740599 | 4 | 159123611 | A | G | 0.252886 | 0.235842 | 0.0512963 | 4.27307E-06 | 21.09538736 |  | A | G | 0.2002 | -0.0094 | 0.0279 | 0.7359 |
|  | rs117537486 | 11 | 94351805 | C | G | 0.03072 | 0.596789 | 0.129736 | 4.22474E-06 | 21.11727615 | Seronegative RA | C | G | 0.03109 | 0.2793 | 0.0986 | 0.00462796 |
|  | rs117827497 | 11 | 105212623 | G | A | 0.030462 | -0.583731 | 0.125147 | 3.09585E-06 | 21.7121043 |  | G | A | 0.01452 | 0.0501 | 0.1371 | 0.715 |
|  | rs118006294 | 16 | 10974221 | C | T | 0.050367 | -0.475295 | 0.101815 | 3.03837E-06 | 21.74804622 |  | C | T | 0.02993 | 0.0676 | 0.0992 | 0.495801 |
|  | rs11858369 | 15 | 90495509 | G | A | 0.068381 | 0.437721 | 0.0864978 | 4.18177E-07 | 25.55653255 |  | G | A | 0.08038 | -0.0396 | 0.0619 | 0.5226 |
|  | rs149747348 | 12 | 98890443 | C | G | 0.013186 | -0.895407 | 0.194003 | 3.92292E-06 | 21.25892825 |  | C | G | 0.04022 | -0.1833 | 0.0837 | 0.0286398 |
|  | rs3998182 | 6 | 32561915 | C | T | 0.27555 | 0.284762 | 0.0548049 | 2.03718E-07 | 26.94279068 |  | C | T | 0.1722 | 0.0557 | 0.0474 | 0.2401 |
|  | rs4268452 | 10 | 81319354 | T | C | 0.071203 | -0.384976 | 0.0840329 | 4.62189E-06 | 20.94526676 |  | T | C | 0.06249 | 0.1335 | 0.068 | 0.0497405 |
|  | rs6530847 | 8 | 15111565 | A | T | 0.391211 | -0.210266 | 0.0452734 | 3.41154E-06 | 21.52628924 |  | A | T | 0.2758 | 0.0161 | 0.0369 | 0.6623 |
|  | rs75170215 | 3 | 25908239 | C | T | 0.02057 | 0.743256 | 0.156416 | 2.01614E-06 | 22.53366242 |  | C | T | 0.02854 | 0.0448 | 0.1003 | 0.655 |
|  | rs75740599 | 4 | 159123611 | A | G | 0.252886 | 0.235842 | 0.0512963 | 4.27307E-06 | 21.09538736 |  | A | G | 0.2002 | -0.007 | 0.0412 | 0.8648 |
| OMP | rs116944686 | 8 | 101374808 | A | G | 0.023319 | 0.417806 | 0.0913422 | 4.78344E-06 | 20.92191891 | Seropositive RA | A | G | 0.01952 | -0.128 | 0.081 | 0.1141 |
|  | rs143477841 | 2 | 24042805 | A | G | 0.012119 | -0.572525 | 0.124655 | 4.3718E-06 | 21.09427528 |  | A | G | 0.002858 | 0.1771 | 0.2162 | 0.4127 |
|  | rs1892331 | 20 | 59326926 | G | A | 0.032321 | 0.376964 | 0.0761458 | 7.40048E-07 | 24.50772233 |  | G | A | 0.01632 | 0.0274 | 0.0907 | 0.7625 |
|  | rs55862931 | 2 | 2672878 | C | G | 0.258412 | -0.146339 | 0.0316679 | 3.81812E-06 | 21.3538772 |  | C | G | 0.2063 | 0.0206 | 0.0277 | 0.4575 |
|  | rs703135 | 1 | 158678095 | A | G | 0.417825 | -0.133184 | 0.0280688 | 2.08593E-06 | 22.51394086 |  | A | G | 0.4905 | -0.0101 | 0.0225 | 0.651799 |
|  | rs8019346 | 14 | 65353011 | A | G | 0.08492 | 0.229272 | 0.0498505 | 4.24141E-06 | 21.15232478 |  | A | G | 0.05806 | -0.0277 | 0.0484 | 0.5675 |
|  | rs116944686 | 8 | 101374808 | A | G | 0.023319 | 0.417806 | 0.0913422 | 4.78344E-06 | 20.92191891 | Seronegative RA | A | G | 0.01955 | -0.0886 | 0.1197 | 0.4592 |
|  | rs143477841 | 2 | 24042805 | A | G | 0.012119 | -0.572525 | 0.124655 | 4.3718E-06 | 21.09427528 |  | A | G | 0.002839 | -0.3686 | 0.3165 | 0.2441 |
|  | rs1892331 | 20 | 59326926 | G | A | 0.032321 | 0.376964 | 0.0761458 | 7.40048E-07 | 24.50772233 |  | G | A | 0.01634 | 0.118 | 0.1325 | 0.373 |
|  | rs55862931 | 2 | 2672878 | C | G | 0.258412 | -0.146339 | 0.0316679 | 3.81812E-06 | 21.3538772 |  | C | G | 0.2063 | 0.0346 | 0.041 | 0.3984 |
|  | rs703135 | 1 | 158678095 | A | G | 0.417825 | -0.133184 | 0.0280688 | 2.08593E-06 | 22.51394086 |  | A | G | 0.4905 | 0.0111 | 0.0332 | 0.736999 |
|  | rs8019346 | 14 | 65353011 | A | G | 0.08492 | 0.229272 | 0.0498505 | 4.24141E-06 | 21.15232478 |  | A | G | 0.0581 | -0.0506 | 0.0704 | 0.4721 |
| IgG | rs10044474 | 5 | 67425603 | G | T | 0.06739 | 0.0653924 | 0.0137102 | 1.84578E-06 | 22.74403549 | Seropositive RA | G | T | 0.1193 | -0.0106 | 0.0349 | 0.7613 |
|  | rs111575036 | 10 | 45194547 | T | C | 0.018243 | -0.116546 | 0.0252727 | 3.99715E-06 | 21.26140753 |  | T | C | 0.00996 | 0.0004 | 0.116 | 0.9972 |
|  | rs11201988 | 10 | 88113475 | A | G | 0.117772 | 0.0505729 | 0.0109411 | 3.79507E-06 | 21.36064154 |  | A | G | 0.1134 | -0.0154 | 0.0358 | 0.6672 |
|  | rs117192929 | 7 | 89122359 | G | A | 0.044981 | 0.0767204 | 0.0166915 | 4.29883E-06 | 21.12182958 |  | G | A | 0.06779 | 0.0085 | 0.0452 | 0.8513 |
|  | rs17232730 | 8 | 129537746 | C | G | 0.121486 | -0.0521098 | 0.0104981 | 6.91497E-07 | 24.63303664 |  | C | G | 0.09601 | -0.0701 | 0.0382 | 0.06608 |
|  | rs60696037 | 1 | 23082564 | T | C | 0.091749 | -0.0561058 | 0.0118003 | 1.98847E-06 | 22.60112058 |  | T | C | 0.1631 | 0.0095 | 0.0305 | 0.7565 |
|  | rs62195865 | 20 | 8239626 | C | T | 0.019004 | 0.117182 | 0.0247662 | 2.22828E-06 | 22.38224325 |  | C | T | 0.01368 | -0.1055 | 0.0989 | 0.2861 |
|  | rs681900 | 2 | 75074967 | C | T | 0.240756 | -0.0375028 | 0.00790877 | 2.11695E-06 | 22.48071123 |  | C | T | 0.179 | -0.0238 | 0.0296 | 0.4221 |
|  | rs74347185 | 12 | 75543807 | A | G | 0.052948 | 0.0780404 | 0.0151369 | 2.52749E-07 | 26.57453296 |  | A | G | 0.0543 | 0.0177 | 0.0499 | 0.7225 |
|  | rs76753623 | 15 | 61751677 | G | A | 0.039382 | 0.0842701 | 0.0172103 | 9.75574E-07 | 23.97014647 |  | G | A | 0.06473 | -0.0331 | 0.0461 | 0.4724 |
|  | rs10044474 | 5 | 67425603 | G | T | 0.06739 | 0.0653924 | 67425603 | 0.0137102 | 22.74403549 | Seronegative RA | G | T | 0.1192 | -0.0635 | 0.0514 | 0.2161 |
|  | rs111575036 | 10 | 45194547 | T | C | 0.018243 | -0.116546 | 45194547 | 0.0252727 | 21.26140753 |  | T | C | 0.01 | 0.3525 | 0.1691 | 0.0370997 |
|  | rs11201988 | 10 | 88113475 | A | G | 0.117772 | 0.0505729 | 88113475 | 0.0109411 | 21.36064154 |  | A | G | 0.1133 | -0.0807 | 0.0531 | 0.1282 |
|  | rs117192929 | 7 | 89122359 | G | A | 0.044981 | 0.0767204 | 89122359 | 0.0166915 | 21.12182958 |  | G | A | 0.06779 | 0.0384 | 0.067 | 0.5662 |
|  | rs60696037 | 1 | 23082564 | T | C | 0.091749 | -0.0561058 | 23082564 | 0.0118003 | 22.60112058 |  | T | C | 0.163 | -0.0185 | 0.0449 | 0.68 |
|  | rs62195865 | 20 | 8239626 | C | T | 0.019004 | 0.117182 | 8239626 | 0.0247662 | 22.38224325 |  | C | T | 0.0137 | -0.1193 | 0.1424 | 0.4024 |
|  | rs681900 | 2 | 75074967 | C | T | 0.240756 | -0.0375028 | 75074967 | 0.00790877 | 22.48071123 |  | C | T | 0.1791 | -0.011 | 0.0437 | 0.8022 |
|  | rs74347185 | 12 | 75543807 | A | G | 0.052948 | 0.0780404 | 75543807 | 0.0151369 | 26.57453296 |  | A | G | 0.05424 | -0.0781 | 0.0739 | 0.2904 |
|  | rs76753623 | 15 | 61751677 | G | A | 0.039382 | 0.0842701 | 61751677 | 0.0172103 | 23.97014647 |  | G | A | 0.06475 | -0.0262 | 0.0684 | 0.701601 |
| UREA | rs117994655 | 19 | 8188056 | A | G | 0.032509 | 0.402018 | 0.0828335 | 1.21409E-06 | 23.53384742 | Seropositive RA | A | G | 0.0284 | -0.1303 | 0.069 | 0.05886 |
|  | rs12820262 | 12 | 129244638 | A | G | 0.177117 | 0.206073 | 0.0393175 | 1.59496E-07 | 27.4463348 |  | A | G | 0.1385 | -0.0088 | 0.0326 | 0.786799 |
|  | rs134748 | 22 | 26543287 | T | G | 0.653005 | -0.142202 | 0.0306515 | 3.49551E-06 | 21.50413137 |  | T | G | 0.637 | 0.0083 | 0.0232 | 0.7204 |
|  | rs143570118 | 17 | 76370080 | T | C | 0.029208 | 0.434126 | 0.0926047 | 2.75962E-06 | 21.95732831 |  | T | C | 0.02283 | 0.0219 | 0.0807 | 0.7857 |
|  | rs4689912 | 4 | 4659633 | C | T | 0.267281 | -0.158009 | 0.0331663 | 1.89657E-06 | 22.67689348 |  | C | T | 0.2533 | 0.0148 | 0.0257 | 0.5648 |
|  | rs60386004 | 14 | 80136052 | T | A | 0.158161 | 0.191768 | 0.0407276 | 2.49482E-06 | 22.15075843 |  | T | A | 0.2275 | 0.0052 | 0.0268 | 0.8449 |
|  | rs61907106 | 12 | 4003258 | A | G | 0.048196 | -0.358847 | 0.0702596 | 3.26543E-07 | 26.06281097 |  | A | G | 0.04879 | 0.0782 | 0.052 | 0.1328 |
|  | rs71569678 | 6 | 23380648 | C | A | 0.060345 | 0.340169 | 0.0613821 | 2.99316E-08 | 30.68456298 |  | C | A | 0.07284 | 0.0075 | 0.0434 | 0.8635 |
|  | rs75477465 | 8 | 68794331 | A | G | 0.034029 | 0.374873 | 0.0802966 | 3.03242E-06 | 21.77649457 |  | A | G | 0.0243 | 0.1024 | 0.0741 | 0.1668 |
|  | rs9289888 | 3 | 152849257 | T | C | 0.066303 | 0.272084 | 0.0573847 | 2.12236E-06 | 22.46093012 |  | T | C | 0.08971 | -0.0003 | 0.0393 | 0.9933 |
|  | rs117994655 | 19 | 8188056 | A | G | 0.032509 | 0.402018 | 0.0828335 | 1.21409E-06 | 23.53384742 | Seronegative RA | A | G | 0.02847 | -0.0472 | 0.1011 | 0.6402 |
|  | rs12820262 | 12 | 129244638 | A | G | 0.177117 | 0.206073 | 0.0393175 | 1.59496E-07 | 27.4463348 |  | A | G | 0.1384 | -0.0321 | 0.0479 | 0.5028 |
|  | rs134748 | 22 | 26543287 | T | G | 0.653005 | -0.142202 | 0.0306515 | 3.49551E-06 | 21.50413137 |  | T | G | 0.637 | -0.0352 | 0.0343 | 0.3046 |
|  | rs143570118 | 17 | 76370080 | T | C | 0.029208 | 0.434126 | 0.0926047 | 2.75962E-06 | 21.95732831 |  | T | C | 0.02286 | 0.0908 | 0.1178 | 0.4409 |
|  | rs4689912 | 4 | 4659633 | C | T | 0.267281 | -0.158009 | 0.0331663 | 1.89657E-06 | 22.67689348 |  | C | T | 0.2532 | -0.0208 | 0.0379 | 0.582599 |
|  | rs60386004 | 14 | 80136052 | T | A | 0.158161 | 0.191768 | 0.0407276 | 2.49482E-06 | 22.15075843 |  | T | A | 0.2276 | 0.0474 | 0.0395 | 0.2306 |
|  | rs61907106 | 12 | 4003258 | A | G | 0.048196 | -0.358847 | 0.0702596 | 3.26543E-07 | 26.06281097 |  | A | G | 0.04872 | 0.0424 | 0.0767 | 0.5807 |
|  | rs71569678 | 6 | 23380648 | C | A | 0.060345 | 0.340169 | 0.0613821 | 2.99316E-08 | 30.68456298 |  | C | A | 0.07285 | 0.0169 | 0.0639 | 0.7913 |
|  | rs75477465 | 8 | 68794331 | A | G | 0.034029 | 0.374873 | 0.0802966 | 3.03242E-06 | 21.77649457 |  | A | G | 0.02424 | -0.0622 | 0.1072 | 0.562 |
|  | rs9289888 | 3 | 152849257 | T | C | 0.066303 | 0.272084 | 0.0573847 | 2.12236E-06 | 22.46093012 |  | T | C | 0.08978 | 0.0649 | 0.0578 | 0.2615 |
| VacA | rs11044935 | 12 | 20062085 | T | A | 0.039367 | 0.428278 | 0.0905091 | 2.22454E-06 | 22.36216242 | Seropositive RA | T | A | 0.03168 | 0.0648 | 0.066 | 0.3261 |
|  | rs113063793 | 1 | 217309978 | T | A | 0.056242 | 0.373402 | 0.075648 | 7.97223E-07 | 24.33353121 |  | T | A | 0.05561 | -0.0825 | 0.0491 | 0.0927791 |
|  | rs113845906 | 3 | 94372336 | A | G | 0.045066 | 0.413478 | 0.0845187 | 9.97401E-07 | 23.90264467 |  | A | G | 0.01478 | -0.1254 | 0.0997 | 0.2088 |
|  | rs117077218 | 10 | 90083333 | T | C | 0.016656 | 0.640222 | 0.138755 | 3.94903E-06 | 21.26232061 |  | T | C | 0.01641 | -0.1385 | 0.0898 | 0.1228 |
|  | rs133537 | 22 | 48595971 | C | T | 0.615079 | -0.173609 | 0.0367088 | 2.25237E-06 | 22.3383378 |  | C | T | 0.5822 | -0.0072 | 0.0229 | 0.753101 |
|  | rs148556020 | 12 | 28990603 | A | T | 0.016883 | 0.63085 | 0.137457 | 4.44427E-06 | 21.03610118 |  | A | T | 0.02866 | 0.0822 | 0.0677 | 0.2249 |
|  | rs1530121 | 5 | 124624902 | C | T | 0.86313 | -0.266111 | 0.0511414 | 1.95637E-07 | 27.04127639 |  | C | T | 0.8995 | -0.026 | 0.0376 | 0.4887 |
|  | rs372744619 | 6 | 32434141 | G | A | 0.010519 | 0.887411 | 0.173802 | 3.29276E-07 | 26.03675369 |  | G | A | 0.02761 | -0.1907 | 0.0689 | 0.00567806 |
|  | rs59104649 | 10 | 129150690 | T | A | 0.014016 | 0.68347 | 0.147207 | 3.43534E-06 | 21.52924239 |  | T | A | 0.03461 | -0.0085 | 0.0616 | 0.8902 |
|  | rs7019543 | 9 | 110365310 | T | G | 0.320078 | 0.18107 | 0.0380994 | 2.00845E-06 | 22.55815046 |  | T | G | 0.2616 | 0.009 | 0.0255 | 0.723299 |
|  | rs72645538 | 1 | 9453174 | G | A | 0.011992 | 0.752792 | 0.164377 | 4.65715E-06 | 20.94665351 |  | G | A | 0.005756 | -0.0023 | 0.1576 | 0.9885 |
|  | rs73500239 | 16 | 1332341 | C | G | 0.144805 | 0.236088 | 0.0500401 | 2.38199E-06 | 22.23096152 |  | C | G | 0.1023 | -0.0155 | 0.0371 | 0.6758 |
|  | rs77497849 | 7 | 32049959 | G | A | 0.119223 | 0.264227 | 0.0538191 | 9.12935E-07 | 24.07289294 |  | G | A | 0.09186 | -0.0227 | 0.0389 | 0.5608 |
|  | rs9606224 | 22 | 20034187 | T | C | 0.029841 | 0.507461 | 0.10223 | 6.90876E-07 | 24.60908004 |  | T | C | 0.04878 | -0.0165 | 0.0528 | 0.7548 |
|  | rs11044935 | 12 | 20062085 | T | A | 0.039367 | 0.428278 | 0.0905091 | 2.22454E-06 | 22.36216242 | Seronegative RA | T | A | 0.03166 | 0.0511 | 0.0974 | 0.599601 |
|  | rs113063793 | 1 | 217309978 | T | A | 0.056242 | 0.373402 | 0.075648 | 7.97223E-07 | 24.33353121 |  | T | A | 0.05575 | 0.0435 | 0.0717 | 0.5443 |
|  | rs113845906 | 3 | 94372336 | A | G | 0.045066 | 0.413478 | 0.0845187 | 9.97401E-07 | 23.90264467 |  | A | G | 0.01485 | 0.0689 | 0.1409 | 0.625 |
|  | rs117077218 | 10 | 90083333 | T | C | 0.016656 | 0.640222 | 0.138755 | 3.94903E-06 | 21.26232061 |  | T | C | 0.01645 | -0.1054 | 0.1312 | 0.4218 |
|  | rs133537 | 22 | 48595971 | C | T | 0.615079 | -0.173609 | 0.0367088 | 2.25237E-06 | 22.3383378 |  | C | T | 0.5822 | -0.015 | 0.0338 | 0.6574 |
|  | rs148556020 | 12 | 28990603 | A | T | 0.016883 | 0.63085 | 0.137457 | 4.44427E-06 | 21.03610118 |  | A | T | 0.02864 | 0.0978 | 0.0998 | 0.327 |
|  | rs1530121 | 5 | 124624902 | C | T | 0.86313 | -0.266111 | 0.0511414 | 1.95637E-07 | 27.04127639 |  | C | T | 0.8996 | 0.0469 | 0.0558 | 0.4007 |
|  | rs372744619 | 6 | 32434141 | G | A | 0.010519 | 0.887411 | 0.173802 | 3.29276E-07 | 26.03675369 |  | G | A | 0.02772 | -0.101 | 0.0997 | 0.3109 |
|  | rs59104649 | 10 | 129150690 | T | A | 0.014016 | 0.68347 | 0.147207 | 3.43534E-06 | 21.52924239 |  | T | A | 0.0346 | 0.0514 | 0.0931 | 0.580801 |
|  | rs7019543 | 9 | 110365310 | T | G | 0.320078 | 0.18107 | 0.0380994 | 2.00845E-06 | 22.55815046 |  | T | G | 0.2616 | 0.0037 | 0.0376 | 0.9212 |
|  | rs72645538 | 1 | 9453174 | G | A | 0.011992 | 0.752792 | 0.164377 | 4.65715E-06 | 20.94665351 |  | G | A | 0.005765 | 0.0465 | 0.2284 | 0.8388 |
|  | rs73500239 | 16 | 1332341 | C | G | 0.144805 | 0.236088 | 0.0500401 | 2.38199E-06 | 22.23096152 |  | C | G | 0.1023 | 0.0163 | 0.0546 | 0.7651 |
|  | rs77497849 | 7 | 32049959 | G | A | 0.119223 | 0.264227 | 0.0538191 | 9.12935E-07 | 24.07289294 |  | G | A | 0.09195 | 0.0568 | 0.0576 | 0.3235 |
|  | rs9606224 | 22 | 20034187 | T | C | 0.029841 | 0.507461 | 0.10223 | 6.90876E-07 | 24.60908004 |  | T | C | 0.04876 | -0.0891 | 0.0781 | 0.2537 |
| Catalase | rs147016571 | 16 | 25837192 | G | A | 0.010184 | -0.839947 | 0.175759 | 1.76202E-06 | 22.80923712 | Seropositive RA | G | A | 0.002441 | -0.3322 | 0.228 | 0.1452 |
|  | rs17647677 | 13 | 46749781 | C | T | 0.041284 | -0.394124 | 0.0851199 | 3.65275E-06 | 21.41143099 |  | C | T | 0.07467 | 0.0327 | 0.0431 | 0.4472 |
|  | rs190890483 | 2 | 157083346 | A | G | 0.012211 | -0.771796 | 0.156854 | 8.63376E-07 | 24.17998291 |  | A | G | 0.04321 | 0.09 | 0.0546 | 0.0992407 |
|  | rs343686 | 6 | 80607062 | C | A | 0.620724 | -0.174703 | 0.036546 | 1.74985E-06 | 22.82249734 |  | C | A | 0.5894 | 0.0093 | 0.0228 | 0.684101 |
|  | rs6456714 | 6 | 26335346 | G | C | 0.631631 | 0.170902 | 0.0360891 | 2.18464E-06 | 22.39671514 |  | G | C | 0.6932 | 0.0102 | 0.0245 | 0.677599 |
|  | rs72808426 | 2 | 58133048 | T | C | 0.035007 | -0.433914 | 0.0938646 | 3.7866E-06 | 21.34252491 |  | T | C | 0.01792 | -0.0317 | 0.0859 | 0.7118 |
|  | rs77162569 | 7 | 148264987 | T | C | 0.025229 | -0.555536 | 0.10878 | 3.27341E-07 | 26.04764386 |  | T | C | 0.03432 | 0.0327 | 0.0607 | 0.5905 |
|  | rs927062 | 14 | 33095049 | G | A | 0.213718 | -0.197935 | 0.0421097 | 2.59591E-06 | 22.06597061 |  | G | A | 0.1403 | -0.0087 | 0.0324 | 0.787601 |
|  | rs147016571 | 16 | 25837192 | G | A | 0.010184 | -0.839947 | 0.175759 | 1.76202E-06 | 22.80923712 | Seronegative RA | G | A | 0.00245 | -0.3985 | 0.3297 | 0.2267 |
|  | rs17647677 | 13 | 46749781 | C | T | 0.041284 | -0.394124 | 0.0851199 | 3.65275E-06 | 21.41143099 |  | C | T | 0.07463 | 0.0231 | 0.0638 | 0.716699 |
|  | rs190890483 | 2 | 157083346 | A | G | 0.012211 | -0.771796 | 0.156854 | 8.63376E-07 | 24.17998291 |  | A | G | 0.04307 | -0.0431 | 0.0809 | 0.5943 |
|  | rs343686 | 6 | 80607062 | C | A | 0.620724 | -0.174703 | 0.036546 | 1.74985E-06 | 22.82249734 |  | C | A | 0.5895 | 0.0851 | 0.0336 | 0.01126 |
|  | rs6456714 | 6 | 26335346 | G | C | 0.631631 | 0.170902 | 0.0360891 | 2.18464E-06 | 22.39671514 |  | G | C | 0.6931 | 0.0183 | 0.0361 | 0.611399 |
|  | rs72808426 | 2 | 58133048 | T | C | 0.035007 | -0.433914 | 0.0938646 | 3.7866E-06 | 21.34252491 |  | T | C | 0.01795 | 0.0293 | 0.1257 | 0.8157 |
|  | rs77162569 | 7 | 148264987 | T | C | 0.025229 | -0.555536 | 0.10878 | 3.27341E-07 | 26.04764386 |  | T | C | 0.03425 | 0.0086 | 0.0917 | 0.9254 |
|  | rs927062 | 14 | 33095049 | G | A | 0.213718 | -0.197935 | 0.0421097 | 2.59591E-06 | 22.06597061 |  | G | A | 0.1404 | 0.0429 | 0.0476 | 0.3684 |
| GroEL | rs117341694 | 13 | 66889675 | A | G | 0.013029 | 0.54338 | 0.115856 | 2.73036E-06 | 21.98114526 | Seropositive RA | A | G | 0.01854 | 0.048 | 0.0828 | 0.562 |
|  | rs1367344 | 18 | 71479072 | C | T | 0.223984 | -0.155199 | 0.0330617 | 2.67621E-06 | 22.01950508 |  | C | T | 0.232 | -0.0129 | 0.027 | 0.6341 |
|  | rs3104037 | 6 | 107139657 | T | C | 0.613619 | 0.134138 | 0.0281607 | 1.90445E-06 | 22.67236576 |  | T | C | 0.6365 | -0.0541 | 0.0233 | 0.02016 |
|  | rs61680606 | 10 | 128952219 | C | A | 0.225838 | 0.152271 | 0.0322746 | 2.38199E-06 | 22.24296818 |  | C | A | 0.3279 | -0.029 | 0.0239 | 0.2248 |
|  | rs117341694 | 13 | 66889675 | A | G | 0.013029 | 0.54338 | 0.115856 | 2.73036E-06 | 21.98114526 | Seronegative RA | A | G | 0.01848 | -0.116 | 0.1251 | 0.3537 |
|  | rs1367344 | 18 | 71479072 | C | T | 0.223984 | -0.155199 | 0.0330617 | 2.67621E-06 | 22.01950508 |  | C | T | 0.2321 | 0.0163 | 0.0399 | 0.6827 |
|  | rs3104037 | 6 | 107139657 | T | C | 0.613619 | 0.134138 | 0.0281607 | 1.90445E-06 | 22.67236576 |  | T | C | 0.6367 | -0.0517 | 0.0344 | 0.1327 |
|  | rs61680606 | 10 | 128952219 | C | A | 0.225838 | 0.152271 | 0.0322746 | 2.38199E-06 | 22.24296818 |  | C | A | 0.328 | 0.0004 | 0.0353 | 0.9901 |
| Allele effects on Exposure: the association summary statistics of the SNP on exposure. Allele effects on Outcome: the association summary statistics of the SNP on outcome. | | | | | | | | | | | | | | | | |  |
