# Supplementary material for: Association between Helicobacter pylori infection and rheumatoid arthritis: Results from the NHANES 1999–2000 and a Mendelian randomization study
Source: Medicine (Baltimore). 2026 Sep 11;105(37):e50458. doi: 10.1097/MD.0000000000050458 (PMC13574391; doi:10.1097/MD.0000000000050458)
Supplement: Supplementary file 3 [file medi-105-e50458-s003.docx]

| Supplementary Table S2 Baseline characteristics distribution of RA and Non-RA patients | | | |
| --- | --- | --- | --- |
| **Characteristics** | Non-RA | RA | p-value |
| **Overall** | 276 | 273 |  |
| **Age (years)** | 62.22(70.31) | 58.23(79.19) | 0.054 |
| **Gender (%)** |  |  | 0.167 |
| Male | 101 (0.34) | 106 (0.41) |  |
| Female | 175 (0.66) | 167 (0.59) |  |
| **Race (%)** | | | <0.001 |
| Mexican American | 36 (0.13) | 71 (0.26) |  |
| Other Hispanic | 11 (0.04) | 11 (0.04) |  |
| Non-Hispanic White | 181 (0.66) | 122 (0.45) |  |
| Non-Hispanic Black | 39 (0.14) | 61 (0.22) |  |
| Other Race | 9 (0.03) | 8 (0.03) |  |
| **BMI** | 29.97（0.73） | 28.71(24.12) | 0.186 |
| **Alcohol(%)** | | | 0.077 |
| No | 183 (0.64) | 149（0.61） |  |
| Yes | 93 (0.36) | 124 (0.39) |  |
| **Smoking(%)** | | | 0.288 |
| No | 152 (0.54) | 147 (0.58) |  |
| Yes | 124 (0.46) | 126 (0.42) |  |
| **Hypertension(%)** | | | 0.351 |
| No | 42 (0.15) | 60 (0.14) |  |
| Yes | 234 (0.85) | 213 (0.86) |  |
| **Diabetes(%)** | | | <0.001 |
| No | 140 (0.47) | 139 (0.46) |  |
| Yes | 136 (0.53) | 134 (0.54) |  |
| Data were presented as mean (SD) or n (%). BMI: body mass index; RA: Rheumatoid arthritis. | | | |
