# Supplementary material for: Association between Helicobacter pylori infection and rheumatoid arthritis: Results from the NHANES 1999–2000 and a Mendelian randomization study
Source: Medicine (Baltimore). 2026 Sep 11;105(37):e50458. doi: 10.1097/MD.0000000000050458 (PMC13574391; doi:10.1097/MD.0000000000050458)
Supplement: Supplementary file 4 [file medi-105-e50458-s007.docx]

| **Supplementary Table S4** Summary statistics of genetic instruments when RA are considered as exposure | | | | | | | | | | | | | | | | | |
| --- | --- | --- | --- | --- | --- | --- | --- | --- | --- | --- | --- | --- | --- | --- | --- | --- | --- |
| **Exposure** | **Instrumental variables of Exposure** | | | **Allele effects on Exposure** | | | | | | | **Allele effects on Outcome** | | | | | | |
|  | **rsid** | **Chromosome** | **Position** | **effect_allele.exposure** | **other_allele.exposure** | **eaf.exposure** | **beta.exposure** | **se.exposure** | **pval.exposure** | ***F*** | **outcome** | **effect_allele.outcome** | **other_allele.outcome** | **eaf.outcome** | **beta.outcome** | **se.outcome** | **pval.outcome** |
| Seropositive RA | rs11571293 | 2 | 204717713 | T | G | 0.3161 | -0.155 | 0.0242 | 1.61002E-10 | 41.02303324 | CagA | T | G | 0.410432 | 0.3161 | 0.0452005 | 0.172415 |
|  | rs116818505 | 6 | 33051900 | G | T | 0.2554 | -0.2795 | 0.0274 | 2.00078E-24 | 104.0537181 |  | G | T | 0.264538 | 0.2554 | 0.051841 | 0.873456 |
|  | rs2523572 | 6 | 31329494 | T | C | 0.6014 | 0.3559 | 0.0256 | 6.25317E-44 | 193.2729831 |  | T | C | 0.644189 | 0.6014 | 0.0484163 | 0.323953 |
|  | rs3117134 | 6 | 32313550 | C | T | 0.5136 | -0.2435 | 0.0232 | 1.16011E-25 | 110.1582596 |  | C | T | 0.372508 | 0.5136 | 0.0450358 | 0.0167571 |
|  | rs34434863 | 6 | 32559673 | G | T | 0.3395 | 0.9684 | 0.0315 | 1E-200 | 945.1126118 |  | G | T | 0.282759 | 0.3395 | 0.0500446 | 0.45248 |
|  | rs34536443 | 19 | 10463118 | C | G | 0.03042 | -0.4029 | 0.0681 | 3.25297E-09 | 35.00220593 |  | C | G | 0.043269 | 0.03042 | 0.111018 | 0.823104 |
|  | rs6679677 | 1 | 114303808 | A | C | 0.1471 | 0.4608 | 0.0325 | 9.47764E-46 | 201.0265056 |  | A | C | 0.102308 | 0.1471 | 0.0722623 | 0.231601 |
|  | rs7731626 | 5 | 55444683 | A | G | 0.2799 | -0.1397 | 0.0251 | 2.46201E-08 | 30.97707975 |  | A | G | 0.380378 | 0.2799 | 0.0467979 | 0.477557 |
|  | rs9264277 | 6 | 31224667 | C | T | 0.7288 | 0.1958 | 0.0261 | 6.61455E-14 | 56.27810493 |  | C | T | 0.633369 | 0.7288 | 0.0469871 | 0.509295 |
|  | rs10885448 | 10 | 115168650 | T | C | 0.6625 | 0.1129 | 0.0239 | 0.000002266 | 22.31450136 | OMP | T | C | 0.703119 | -0.0578086 | 0.031025 | 0.0624209 |
|  | rs11571293 | 2 | 204717713 | T | G | 0.3161 | -0.155 | 0.0242 | 1.61002E-10 | 41.02303324 |  | T | G | 0.394408 | -0.0221052 | 0.0287296 | 0.441642 |
|  | rs116234576 | 4 | 190172971 | C | T | 0.02789 | 0.3716 | 0.0718 | 2.306E-07 | 26.78536857 |  | C | T | 0.022543 | 0.0941945 | 0.0951982 | 0.32244 |
|  | rs116844046 | 7 | 53328220 | T | G | 0.08912 | -0.1873 | 0.0397 | 2.30701E-06 | 22.25817978 |  | T | G | 0.066211 | -0.00622608 | 0.0547939 | 0.909533 |
|  | rs11699962 | 20 | 5417059 | A | G | 0.07591 | 0.2075 | 0.0429 | 0.000001286 | 23.39465916 |  | A | G | 0.05272 | -0.0613239 | 0.064724 | 0.3434 |
|  | rs12982466 | 19 | 11297289 | T | C | 0.05141 | -0.2414 | 0.0514 | 0.000002651 | 22.05684535 |  | T | C | 0.090167 | 0.0424756 | 0.0483718 | 0.379887 |
|  | rs1489318 | 15 | 63260449 | G | T | 0.8557 | 0.1695 | 0.0322 | 1.47001E-07 | 27.70912209 |  | G | T | 0.857143 | -0.00539822 | 0.0390696 | 0.890107 |
|  | rs1571878 | 6 | 167540842 | T | C | 0.5532 | -0.1355 | 0.0227 | 2.23599E-09 | 35.63050523 |  | T | C | 0.569369 | -0.0368153 | 0.0279041 | 0.18705 |
|  | rs2476601 | 1 | 114377568 | G | A | 0.8515 | -0.4652 | 0.0323 | 5.48277E-47 | 207.4289992 |  | G | A | 0.901282 | 0.0331381 | 0.0470769 | 0.481486 |
|  | rs34434863 | 6 | 32559673 | G | T | 0.3395 | 0.9684 | 0.0315 | 1E-200 | 945.1126118 |  | G | T | 0.290426 | 0.0227238 | 0.0314112 | 0.469416 |
|  | rs34536443 | 19 | 10463118 | C | G | 0.03042 | -0.4029 | 0.0681 | 3.25297E-09 | 35.00220593 |  | C | G | 0.038005 | 0.0333787 | 0.0726665 | 0.645989 |
|  | rs34892989 | 6 | 33799390 | A | G | 0.2073 | -0.2116 | 0.0292 | 4.57826E-13 | 52.51226227 |  | A | G | 0.251587 | 0.0246797 | 0.0321341 | 0.442473 |
|  | rs4073285 | 1 | 2539796 | T | C | 0.3623 | -0.1152 | 0.0233 | 7.48893E-07 | 24.44489751 |  | T | C | 0.343383 | -0.0210353 | 0.0292086 | 0.471418 |
|  | rs62395272 | 6 | 31394424 | T | C | 0.1068 | 0.4882 | 0.0374 | 6.68652E-39 | 170.3913133 |  | T | C | 0.087078 | 0.0236103 | 0.0487634 | 0.628257 |
|  | rs6568622 | 6 | 110298307 | C | A | 0.7893 | -0.1293 | 0.0274 | 2.31201E-06 | 22.26850331 |  | C | A | 0.756942 | 0.0574071 | 0.0324139 | 0.0765508 |
|  | rs7137828 | 12 | 111932800 | T | C | 0.5857 | -0.1138 | 0.0227 | 5.32194E-07 | 25.13204996 |  | T | C | 0.515601 | -0.00576327 | 0.027793 | 0.835726 |
|  | rs72976823 | 6 | 138019512 | A | G | 0.3294 | -0.1242 | 0.0239 | 1.92899E-07 | 27.00489508 |  | A | G | 0.230892 | 0.0588114 | 0.0331645 | 0.0761746 |
|  | rs771289 | 2 | 7066555 | T | G | 0.2976 | -0.1125 | 0.0246 | 4.75094E-06 | 20.9136548 |  | T | G | 0.241338 | -0.0325991 | 0.0334068 | 0.329152 |
|  | rs7731626 | 5 | 55444683 | A | G | 0.2799 | -0.1397 | 0.0251 | 2.46201E-08 | 30.97707975 |  | A | G | 0.38049 | 0.024314 | 0.0290486 | 0.402586 |
|  | rs78222069 | 4 | 123213720 | T | C | 0.01602 | 0.4331 | 0.0923 | 2.70601E-06 | 22.01750329 |  | T | C | 0.021807 | 0.173507 | 0.0959756 | 0.0706334 |
|  | rs78795325 | 10 | 71598195 | G | A | 0.04199 | 0.2595 | 0.0566 | 4.49097E-06 | 21.02020594 |  | G | A | 0.058515 | -0.0483165 | 0.0581201 | 0.405792 |
|  | rs7898906 | 10 | 63815937 | C | T | 0.5048 | -0.1053 | 0.0226 | 3.13603E-06 | 21.70875757 |  | C | T | 0.638833 | -0.0233396 | 0.0292257 | 0.424523 |
|  | rs8179673 | 2 | 191969341 | T | C | 0.7682 | -0.1361 | 0.0266 | 3.19897E-07 | 26.17870033 |  | T | C | 0.776695 | 0.00164373 | 0.0334667 | 0.960827 |
|  | rs9261601 | 6 | 30216146 | G | A | 0.4379 | 0.23 | 0.0243 | 2.48714E-21 | 89.58560468 |  | G | A | 0.470379 | 0.0140653 | 0.028154 | 0.617366 |
|  | rs9272320 | 6 | 32604124 | A | G | 0.4287 | 0.474 | 0.0246 | 6.23735E-83 | 371.262918 |  | A | G | 0.578865 | 0.0399461 | 0.027761 | 0.15017 |
|  | rs9277516 | 6 | 33054280 | C | T | 0.2548 | -0.278 | 0.0275 | 4.15528E-24 | 102.1925671 |  | C | T | 0.30676 | -0.00330935 | 0.0299055 | 0.911886 |
|  | rs932036 | 4 | 26090862 | T | A | 0.283 | 0.1338 | 0.0248 | 7.04904E-08 | 29.10743724 |  | T | A | 0.304721 | 0.0281072 | 0.0295874 | 0.342127 |
|  | rs10885448 | 10 | 115168650 | T | C | 0.6625 | 0.1129 | 0.0239 | 0.000002266 | 22.31450136 | IgG | T | C | 0.701625 | -0.00323728 | 0.00758083 | 0.669354 |
|  | rs11571293 | 2 | 204717713 | T | G | 0.3161 | -0.155 | 0.0242 | 1.61002E-10 | 41.02303324 |  | T | G | 0.405907 | -0.0166674 | 0.00696838 | 0.0167629 |
|  | rs116234576 | 4 | 190172971 | C | T | 0.02789 | 0.3716 | 0.0718 | 2.306E-07 | 26.78536857 |  | C | T | 0.026112 | -0.0467921 | 0.0217181 | 0.031199 |
|  | rs116844046 | 7 | 53328220 | T | G | 0.08912 | -0.1873 | 0.0397 | 2.30701E-06 | 22.25817978 |  | T | G | 0.069917 | -0.000178063 | 0.0131854 | 0.989225 |
|  | rs11699962 | 20 | 5417059 | A | G | 0.07591 | 0.2075 | 0.0429 | 0.000001286 | 23.39465916 |  | A | G | 0.054221 | -0.0294252 | 0.0155013 | 0.0576647 |
|  | rs12982466 | 19 | 11297289 | T | C | 0.05141 | -0.2414 | 0.0514 | 0.000002651 | 22.05684535 |  | T | C | 0.090071 | -0.00513745 | 0.0118652 | 0.665025 |
|  | rs1489318 | 15 | 63260449 | G | T | 0.8557 | 0.1695 | 0.0322 | 1.47001E-07 | 27.70912209 |  | G | T | 0.859555 | -0.00692435 | 0.00977557 | 0.478739 |
|  | rs1571878 | 6 | 167540842 | T | C | 0.5532 | -0.1355 | 0.0227 | 2.23599E-09 | 35.63050523 |  | T | C | 0.559205 | 0.00337393 | 0.00679309 | 0.619421 |
|  | rs2476601 | 1 | 114377568 | G | A | 0.8515 | -0.4652 | 0.0323 | 5.48277E-47 | 207.4289992 |  | G | A | 0.902003 | 0.0023267 | 0.0113749 | 0.837927 |
|  | rs34434863 | 6 | 32559673 | G | T | 0.3395 | 0.9684 | 0.0315 | 1E-200 | 945.1126118 |  | G | T | 0.292884 | -0.00563388 | 0.00773865 | 0.466602 |
|  | rs34536443 | 19 | 10463118 | C | G | 0.03042 | -0.4029 | 0.0681 | 3.25297E-09 | 35.00220593 |  | C | G | 0.040452 | -0.0123564 | 0.0173128 | 0.475405 |
|  | rs34892989 | 6 | 33799390 | A | G | 0.2073 | -0.2116 | 0.0292 | 4.57826E-13 | 52.51226227 |  | A | G | 0.260115 | 0.000558922 | 0.00780699 | 0.942926 |
|  | rs4073285 | 1 | 2539796 | T | C | 0.3623 | -0.1152 | 0.0233 | 7.48893E-07 | 24.44489751 |  | T | C | 0.336677 | -0.00742951 | 0.0071613 | 0.299524 |
|  | rs62395272 | 6 | 31394424 | T | C | 0.1068 | 0.4882 | 0.0374 | 6.68652E-39 | 170.3913133 |  | T | C | 0.091101 | 0.00226951 | 0.0118035 | 0.847527 |
|  | rs6568622 | 6 | 110298307 | C | A | 0.7893 | -0.1293 | 0.0274 | 2.31201E-06 | 22.26850331 |  | C | A | 0.750288 | 0.013445 | 0.00785634 | 0.0870142 |
|  | rs7137828 | 12 | 111932800 | T | C | 0.5857 | -0.1138 | 0.0227 | 5.32194E-07 | 25.13204996 |  | T | C | 0.515496 | -0.00615583 | 0.00673976 | 0.361053 |
|  | rs72976823 | 6 | 138019512 | A | G | 0.3294 | -0.1242 | 0.0239 | 1.92899E-07 | 27.00489508 |  | A | G | 0.225529 | -0.00208714 | 0.00820761 | 0.799269 |
|  | rs771289 | 2 | 7066555 | T | G | 0.2976 | -0.1125 | 0.0246 | 4.75094E-06 | 20.9136548 |  | T | G | 0.234518 | 0.00602682 | 0.00808216 | 0.455853 |
|  | rs7731626 | 5 | 55444683 | A | G | 0.2799 | -0.1397 | 0.0251 | 2.46201E-08 | 30.97707975 |  | A | G | 0.380481 | -0.00581869 | 0.00702778 | 0.407696 |
|  | rs78222069 | 4 | 123213720 | T | C | 0.01602 | 0.4331 | 0.0923 | 2.70601E-06 | 22.01750329 |  | T | C | 0.022643 | -0.0132442 | 0.0230314 | 0.565257 |
|  | rs78795325 | 10 | 71598195 | G | A | 0.04199 | 0.2595 | 0.0566 | 4.49097E-06 | 21.02020594 |  | G | A | 0.060653 | -0.0267942 | 0.0141093 | 0.0575586 |
|  | rs7898906 | 10 | 63815937 | C | T | 0.5048 | -0.1053 | 0.0226 | 3.13603E-06 | 21.70875757 |  | C | T | 0.633679 | 0.0105872 | 0.0071779 | 0.14022 |
|  | rs8179673 | 2 | 191969341 | T | C | 0.7682 | -0.1361 | 0.0266 | 3.19897E-07 | 26.17870033 |  | T | C | 0.775356 | -0.00394269 | 0.00809767 | 0.626335 |
|  | rs9261601 | 6 | 30216146 | G | A | 0.4379 | 0.23 | 0.0243 | 2.48714E-21 | 89.58560468 |  | G | A | 0.474027 | -0.00350151 | 0.00682161 | 0.607743 |
|  | rs9272320 | 6 | 32604124 | A | G | 0.4287 | 0.474 | 0.0246 | 6.23735E-83 | 371.262918 |  | A | G | 0.586916 | 0.00980213 | 0.00690336 | 0.155634 |
|  | rs9277516 | 6 | 33054280 | C | T | 0.2548 | -0.278 | 0.0275 | 4.15528E-24 | 102.1925671 |  | C | T | 0.302001 | 0.0106693 | 0.00739839 | 0.14927 |
|  | rs932036 | 4 | 26090862 | T | A | 0.283 | 0.1338 | 0.0248 | 7.04904E-08 | 29.10743724 |  | T | A | 0.303013 | 0.00677587 | 0.00731259 | 0.354133 |
|  | rs10885448 | 10 | 115168650 | T | C | 0.6625 | 0.1129 | 0.0239 | 0.000002266 | 22.31450136 | UREA | T | C | 0.697987 | -0.0166817 | 0.0333539 | 0.616976 |
|  | rs11571293 | 2 | 204717713 | T | G | 0.3161 | -0.155 | 0.0242 | 1.61002E-10 | 41.02303324 |  | T | G | 0.39387 | 0.0163432 | 0.031058 | 0.598738 |
|  | rs116234576 | 4 | 190172971 | C | T | 0.02789 | 0.3716 | 0.0718 | 2.306E-07 | 26.78536857 |  | C | T | 0.022321 | -0.0358219 | 0.100771 | 0.722232 |
|  | rs116844046 | 7 | 53328220 | T | G | 0.08912 | -0.1873 | 0.0397 | 2.30701E-06 | 22.25817978 |  | T | G | 0.064575 | -0.0647418 | 0.0597598 | 0.278646 |
|  | rs11699962 | 20 | 5417059 | A | G | 0.07591 | 0.2075 | 0.0429 | 0.000001286 | 23.39465916 |  | A | G | 0.049092 | -0.0296198 | 0.0722448 | 0.681813 |
|  | rs12982466 | 19 | 11297289 | T | C | 0.05141 | -0.2414 | 0.0514 | 0.000002651 | 22.05684535 |  | T | C | 0.08961 | -0.00815026 | 0.0514023 | 0.874017 |
|  | rs1489318 | 15 | 63260449 | G | T | 0.8557 | 0.1695 | 0.0322 | 1.47001E-07 | 27.70912209 |  | G | T | 0.857403 | 0.00939613 | 0.042002 | 0.822986 |
|  | rs1571878 | 6 | 167540842 | T | C | 0.5532 | -0.1355 | 0.0227 | 2.23599E-09 | 35.63050523 |  | T | C | 0.563211 | 0.00299961 | 0.0298384 | 0.919925 |
|  | rs2476601 | 1 | 114377568 | G | A | 0.8515 | -0.4652 | 0.0323 | 5.48277E-47 | 207.4289992 |  | G | A | 0.908349 | 0.00682744 | 0.0512803 | 0.894083 |
|  | rs34434863 | 6 | 32559673 | G | T | 0.3395 | 0.9684 | 0.0315 | 1E-200 | 945.1126118 |  | G | T | 0.289578 | -0.0572377 | 0.0340054 | 0.0923379 |
|  | rs34536443 | 19 | 10463118 | C | G | 0.03042 | -0.4029 | 0.0681 | 3.25297E-09 | 35.00220593 |  | C | G | 0.038319 | 0.163953 | 0.0771664 | 0.033614 |
|  | rs34892989 | 6 | 33799390 | A | G | 0.2073 | -0.2116 | 0.0292 | 4.57826E-13 | 52.51226227 |  | A | G | 0.264801 | -0.00405805 | 0.0340789 | 0.905213 |
|  | rs4073285 | 1 | 2539796 | T | C | 0.3623 | -0.1152 | 0.0233 | 7.48893E-07 | 24.44489751 |  | T | C | 0.33798 | 0.0427877 | 0.0316272 | 0.176094 |
|  | rs62395272 | 6 | 31394424 | T | C | 0.1068 | 0.4882 | 0.0374 | 6.68652E-39 | 170.3913133 |  | T | C | 0.09013 | -0.0135768 | 0.051927 | 0.793738 |
|  | rs6568622 | 6 | 110298307 | C | A | 0.7893 | -0.1293 | 0.0274 | 2.31201E-06 | 22.26850331 |  | C | A | 0.750114 | -0.0488528 | 0.0345862 | 0.157804 |
|  | rs7137828 | 12 | 111932800 | T | C | 0.5857 | -0.1138 | 0.0227 | 5.32194E-07 | 25.13204996 |  | T | C | 0.521818 | 0.00704363 | 0.0301614 | 0.815349 |
|  | rs72976823 | 6 | 138019512 | A | G | 0.3294 | -0.1242 | 0.0239 | 1.92899E-07 | 27.00489508 |  | A | G | 0.21392 | 0.0878297 | 0.036264 | 0.0154373 |
|  | rs771289 | 2 | 7066555 | T | G | 0.2976 | -0.1125 | 0.0246 | 4.75094E-06 | 20.9136548 |  | T | G | 0.235076 | 0.00359842 | 0.0358125 | 0.919964 |
|  | rs7731626 | 5 | 55444683 | A | G | 0.2799 | -0.1397 | 0.0251 | 2.46201E-08 | 30.97707975 |  | A | G | 0.385662 | 0.000959303 | 0.0307345 | 0.9751 |
|  | rs78222069 | 4 | 123213720 | T | C | 0.01602 | 0.4331 | 0.0923 | 2.70601E-06 | 22.01750329 |  | T | C | 0.021243 | 0.0274604 | 0.103954 | 0.791657 |
|  | rs78795325 | 10 | 71598195 | G | A | 0.04199 | 0.2595 | 0.0566 | 4.49097E-06 | 21.02020594 |  | G | A | 0.060854 | 0.0166389 | 0.0618708 | 0.787983 |
|  | rs7898906 | 10 | 63815937 | C | T | 0.5048 | -0.1053 | 0.0226 | 3.13603E-06 | 21.70875757 |  | C | T | 0.641892 | 0.0280707 | 0.031554 | 0.373676 |
|  | rs8179673 | 2 | 191969341 | T | C | 0.7682 | -0.1361 | 0.0266 | 3.19897E-07 | 26.17870033 |  | T | C | 0.774613 | -0.0322677 | 0.0357855 | 0.367217 |
|  | rs9261601 | 6 | 30216146 | G | A | 0.4379 | 0.23 | 0.0243 | 2.48714E-21 | 89.58560468 |  | G | A | 0.474471 | 0.0443121 | 0.0295985 | 0.134366 |
|  | rs9272320 | 6 | 32604124 | A | G | 0.4287 | 0.474 | 0.0246 | 6.23735E-83 | 371.262918 |  | A | G | 0.59808 | 0.053074 | 0.0299282 | 0.0761676 |
|  | rs9277516 | 6 | 33054280 | C | T | 0.2548 | -0.278 | 0.0275 | 4.15528E-24 | 102.1925671 |  | C | T | 0.307306 | 0.0443109 | 0.0318345 | 0.163948 |
|  | rs932036 | 4 | 26090862 | T | A | 0.283 | 0.1338 | 0.0248 | 7.04904E-08 | 29.10743724 |  | T | A | 0.307062 | 0.00862222 | 0.0319588 | 0.787319 |
|  | rs10885448 | 10 | 115168650 | T | C | 0.6625 | 0.1129 | 0.0239 | 0.000002266 | 22.31450136 | VacA | T | C | 0.686593 | 0.0230985 | 0.0390639 | 0.554318 |
|  | rs11571293 | 2 | 204717713 | T | G | 0.3161 | -0.155 | 0.0242 | 1.61002E-10 | 41.02303324 |  | T | G | 0.39902 | 0.0830628 | 0.0370013 | 0.0247771 |
|  | rs116234576 | 4 | 190172971 | C | T | 0.02789 | 0.3716 | 0.0718 | 2.306E-07 | 26.78536857 |  | C | T | 0.023633 | 0.188192 | 0.118264 | 0.111544 |
|  | rs116844046 | 7 | 53328220 | T | G | 0.08912 | -0.1873 | 0.0397 | 2.30701E-06 | 22.25817978 |  | T | G | 0.065189 | -0.0665458 | 0.0693582 | 0.337331 |
|  | rs11699962 | 20 | 5417059 | A | G | 0.07591 | 0.2075 | 0.0429 | 0.000001286 | 23.39465916 |  | A | G | 0.048985 | 0.0552561 | 0.08528 | 0.517026 |
|  | rs12982466 | 19 | 11297289 | T | C | 0.05141 | -0.2414 | 0.0514 | 0.000002651 | 22.05684535 |  | T | C | 0.08793 | -0.0810397 | 0.0628518 | 0.197267 |
|  | rs1489318 | 15 | 63260449 | G | T | 0.8557 | 0.1695 | 0.0322 | 1.47001E-07 | 27.70912209 |  | G | T | 0.850654 | 0.0330305 | 0.0495084 | 0.504664 |
|  | rs1571878 | 6 | 167540842 | T | C | 0.5532 | -0.1355 | 0.0227 | 2.23599E-09 | 35.63050523 |  | T | C | 0.568551 | 0.0233567 | 0.0359578 | 0.515978 |
|  | rs2476601 | 1 | 114377568 | G | A | 0.8515 | -0.4652 | 0.0323 | 5.48277E-47 | 207.4289992 |  | G | A | 0.904283 | -0.0589402 | 0.0609924 | 0.333868 |
|  | rs34434863 | 6 | 32559673 | G | T | 0.3395 | 0.9684 | 0.0315 | 1E-200 | 945.1126118 |  | G | T | 0.267767 | -0.0281519 | 0.0415783 | 0.498354 |
|  | rs34536443 | 19 | 10463118 | C | G | 0.03042 | -0.4029 | 0.0681 | 3.25297E-09 | 35.00220593 |  | C | G | 0.040782 | -0.0238577 | 0.08919 | 0.789089 |
|  | rs34892989 | 6 | 33799390 | A | G | 0.2073 | -0.2116 | 0.0292 | 4.57826E-13 | 52.51226227 |  | A | G | 0.268543 | -0.0414902 | 0.0409523 | 0.310996 |
|  | rs4073285 | 1 | 2539796 | T | C | 0.3623 | -0.1152 | 0.0233 | 7.48893E-07 | 24.44489751 |  | T | C | 0.334646 | 0.0736438 | 0.0378713 | 0.0518251 |
|  | rs62395272 | 6 | 31394424 | T | C | 0.1068 | 0.4882 | 0.0374 | 6.68652E-39 | 170.3913133 |  | T | C | 0.085885 | 0.0296296 | 0.0641677 | 0.644258 |
|  | rs6568622 | 6 | 110298307 | C | A | 0.7893 | -0.1293 | 0.0274 | 2.31201E-06 | 22.26850331 |  | C | A | 0.760941 | -0.00301759 | 0.0416021 | 0.942176 |
|  | rs7137828 | 12 | 111932800 | T | C | 0.5857 | -0.1138 | 0.0227 | 5.32194E-07 | 25.13204996 |  | T | C | 0.514658 | -0.00422322 | 0.0355445 | 0.905422 |
|  | rs72976823 | 6 | 138019512 | A | G | 0.3294 | -0.1242 | 0.0239 | 1.92899E-07 | 27.00489508 |  | A | G | 0.237716 | 0.0181581 | 0.0422281 | 0.667195 |
|  | rs76732127 | 18 | 43964431 | A | G | 0.02196 | 0.3566 | 0.0775 | 4.15203E-06 | 21.17163398 |  | A | G | 0.010224 | -0.16512 | 0.17238 | 0.338121 |
|  | rs771289 | 2 | 7066555 | T | G | 0.2976 | -0.1125 | 0.0246 | 4.75094E-06 | 20.9136548 |  | T | G | 0.239576 | 0.0343815 | 0.0422411 | 0.415683 |
|  | rs7731626 | 5 | 55444683 | A | G | 0.2799 | -0.1397 | 0.0251 | 2.46201E-08 | 30.97707975 |  | A | G | 0.386113 | 0.0344631 | 0.037199 | 0.35421 |
|  | rs78222069 | 4 | 123213720 | T | C | 0.01602 | 0.4331 | 0.0923 | 2.70601E-06 | 22.01750329 |  | T | C | 0.024231 | 0.131442 | 0.11523 | 0.254 |
|  | rs78795325 | 10 | 71598195 | G | A | 0.04199 | 0.2595 | 0.0566 | 4.49097E-06 | 21.02020594 |  | G | A | 0.059053 | 0.0970996 | 0.074219 | 0.190777 |
|  | rs7898906 | 10 | 63815937 | C | T | 0.5048 | -0.1053 | 0.0226 | 3.13603E-06 | 21.70875757 |  | C | T | 0.642857 | 0.0405833 | 0.0374475 | 0.278481 |
|  | rs8179673 | 2 | 191969341 | T | C | 0.7682 | -0.1361 | 0.0266 | 3.19897E-07 | 26.17870033 |  | T | C | 0.771512 | 0.0320128 | 0.0429015 | 0.455551 |
|  | rs9261601 | 6 | 30216146 | G | A | 0.4379 | 0.23 | 0.0243 | 2.48714E-21 | 89.58560468 |  | G | A | 0.474571 | 0.0169211 | 0.0356969 | 0.635486 |
|  | rs9272320 | 6 | 32604124 | A | G | 0.4287 | 0.474 | 0.0246 | 6.23735E-83 | 371.262918 |  | A | G | 0.593403 | 0.0311228 | 0.0356085 | 0.382103 |
|  | rs9277516 | 6 | 33054280 | C | T | 0.2548 | -0.278 | 0.0275 | 4.15528E-24 | 102.1925671 |  | C | T | 0.299413 | 0.0362963 | 0.0385768 | 0.346764 |
|  | rs932036 | 4 | 26090862 | T | A | 0.283 | 0.1338 | 0.0248 | 7.04904E-08 | 29.10743724 |  | T | A | 0.310749 | -0.0133533 | 0.0385027 | 0.728731 |
|  | rs10885448 | 10 | 115168650 | T | C | 0.6625 | 0.1129 | 0.0239 | 0.000002266 | 22.31450136 | Catalase | T | C | 0.695232 | -0.0466759 | 0.0386627 | 0.227333 |
|  | rs11571293 | 2 | 204717713 | T | G | 0.3161 | -0.155 | 0.0242 | 1.61002E-10 | 41.02303324 |  | T | G | 0.389585 | -0.0426611 | 0.0369443 | 0.248197 |
|  | rs116234576 | 4 | 190172971 | C | T | 0.02789 | 0.3716 | 0.0718 | 2.306E-07 | 26.78536857 |  | C | T | 0.024265 | 0.00805903 | 0.117003 | 0.945086 |
|  | rs116844046 | 7 | 53328220 | T | G | 0.08912 | -0.1873 | 0.0397 | 2.30701E-06 | 22.25817978 |  | T | G | 0.071241 | -0.0246367 | 0.0660741 | 0.709249 |
|  | rs11699962 | 20 | 5417059 | A | G | 0.07591 | 0.2075 | 0.0429 | 0.000001286 | 23.39465916 |  | A | G | 0.055241 | -0.0934216 | 0.0810662 | 0.249152 |
|  | rs12982466 | 19 | 11297289 | T | C | 0.05141 | -0.2414 | 0.0514 | 0.000002651 | 22.05684535 |  | T | C | 0.085518 | 0.0828037 | 0.0634353 | 0.191782 |
|  | rs1489318 | 15 | 63260449 | G | T | 0.8557 | 0.1695 | 0.0322 | 1.47001E-07 | 27.70912209 |  | G | T | 0.861807 | 0.0296572 | 0.0505061 | 0.557069 |
|  | rs1571878 | 6 | 167540842 | T | C | 0.5532 | -0.1355 | 0.0227 | 2.23599E-09 | 35.63050523 |  | T | C | 0.559539 | 0.0176686 | 0.0359791 | 0.62337 |
|  | rs2476601 | 1 | 114377568 | G | A | 0.8515 | -0.4652 | 0.0323 | 5.48277E-47 | 207.4289992 |  | G | A | 0.90367 | 0.0403704 | 0.0612959 | 0.510143 |
|  | rs34434863 | 6 | 32559673 | G | T | 0.3395 | 0.9684 | 0.0315 | 1E-200 | 945.1126118 |  | G | T | 0.302469 | 0.0354396 | 0.0399236 | 0.37471 |
|  | rs34536443 | 19 | 10463118 | C | G | 0.03042 | -0.4029 | 0.0681 | 3.25297E-09 | 35.00220593 |  | C | G | 0.042282 | 0.0738432 | 0.0885627 | 0.404396 |
|  | rs34892989 | 6 | 33799390 | A | G | 0.2073 | -0.2116 | 0.0292 | 4.57826E-13 | 52.51226227 |  | A | G | 0.252667 | 0.0459463 | 0.0420941 | 0.275047 |
|  | rs4073285 | 1 | 2539796 | T | C | 0.3623 | -0.1152 | 0.0233 | 7.48893E-07 | 24.44489751 |  | T | C | 0.328808 | -0.0153222 | 0.0382136 | 0.688449 |
|  | rs62395272 | 6 | 31394424 | T | C | 0.1068 | 0.4882 | 0.0374 | 6.68652E-39 | 170.3913133 |  | T | C | 0.096633 | 0.0257894 | 0.0598791 | 0.666693 |
|  | rs6568622 | 6 | 110298307 | C | A | 0.7893 | -0.1293 | 0.0274 | 2.31201E-06 | 22.26850331 |  | C | A | 0.758905 | -0.0389037 | 0.0418052 | 0.352064 |
|  | rs7137828 | 12 | 111932800 | T | C | 0.5857 | -0.1138 | 0.0227 | 5.32194E-07 | 25.13204996 |  | T | C | 0.501645 | 0.0276347 | 0.0354594 | 0.435784 |
|  | rs72976823 | 6 | 138019512 | A | G | 0.3294 | -0.1242 | 0.0239 | 1.92899E-07 | 27.00489508 |  | A | G | 0.235274 | 0.0792229 | 0.04178 | 0.0579349 |
|  | rs771289 | 2 | 7066555 | T | G | 0.2976 | -0.1125 | 0.0246 | 4.75094E-06 | 20.9136548 |  | T | G | 0.241437 | 0.0422704 | 0.0418302 | 0.312245 |
|  | rs7731626 | 5 | 55444683 | A | G | 0.2799 | -0.1397 | 0.0251 | 2.46201E-08 | 30.97707975 |  | A | G | 0.374836 | 0.0252526 | 0.0374043 | 0.499596 |
|  | rs78222069 | 4 | 123213720 | T | C | 0.01602 | 0.4331 | 0.0923 | 2.70601E-06 | 22.01750329 |  | T | C | 0.023479 | 0.0878555 | 0.118603 | 0.458845 |
|  | rs78795325 | 10 | 71598195 | G | A | 0.04199 | 0.2595 | 0.0566 | 4.49097E-06 | 21.02020594 |  | G | A | 0.06168 | 0.0396069 | 0.0733783 | 0.58936 |
|  | rs7898906 | 10 | 63815937 | C | T | 0.5048 | -0.1053 | 0.0226 | 3.13603E-06 | 21.70875757 |  | C | T | 0.631809 | 0.0337794 | 0.0377828 | 0.3713 |
|  | rs8179673 | 2 | 191969341 | T | C | 0.7682 | -0.1361 | 0.0266 | 3.19897E-07 | 26.17870033 |  | T | C | 0.778618 | 0.0241861 | 0.0423408 | 0.567847 |
|  | rs9261601 | 6 | 30216146 | G | A | 0.4379 | 0.23 | 0.0243 | 2.48714E-21 | 89.58560468 |  | G | A | 0.466689 | 0.0181313 | 0.034951 | 0.603925 |
|  | rs9272320 | 6 | 32604124 | A | G | 0.4287 | 0.474 | 0.0246 | 6.23735E-83 | 371.262918 |  | A | G | 0.599802 | -0.00199847 | 0.037027 | 0.956956 |
|  | rs9277516 | 6 | 33054280 | C | T | 0.2548 | -0.278 | 0.0275 | 4.15528E-24 | 102.1925671 |  | C | T | 0.305665 | 0.044429 | 0.0389629 | 0.254165 |
|  | rs932036 | 4 | 26090862 | T | A | 0.283 | 0.1338 | 0.0248 | 7.04904E-08 | 29.10743724 |  | T | A | 0.317314 | 0.0369402 | 0.0379649 | 0.330548 |
|  | rs10885448 | 10 | 115168650 | T | C | 0.6625 | 0.1129 | 0.0239 | 0.000002266 | 22.31450136 | GroEL | T | C | 0.692692 | -0.000272655 | 0.0296231 | 0.992656 |
|  | rs11571293 | 2 | 204717713 | T | G | 0.3161 | -0.155 | 0.0242 | 1.61002E-10 | 41.02303324 |  | T | G | 0.395825 | -0.00602978 | 0.0282374 | 0.830907 |
|  | rs116234576 | 4 | 190172971 | C | T | 0.02789 | 0.3716 | 0.0718 | 2.306E-07 | 26.78536857 |  | C | T | 0.026481 | 0.0155089 | 0.0851383 | 0.855456 |
|  | rs116844046 | 7 | 53328220 | T | G | 0.08912 | -0.1873 | 0.0397 | 2.30701E-06 | 22.25817978 |  | T | G | 0.071808 | -0.0116909 | 0.050922 | 0.818414 |
|  | rs11699962 | 20 | 5417059 | A | G | 0.07591 | 0.2075 | 0.0429 | 0.000001286 | 23.39465916 |  | A | G | 0.048761 | -0.0597913 | 0.0652327 | 0.35936 |
|  | rs12982466 | 19 | 11297289 | T | C | 0.05141 | -0.2414 | 0.0514 | 0.000002651 | 22.05684535 |  | T | C | 0.090875 | 0.0195951 | 0.0466908 | 0.674721 |
|  | rs1489318 | 15 | 63260449 | G | T | 0.8557 | 0.1695 | 0.0322 | 1.47001E-07 | 27.70912209 |  | G | T | 0.852863 | 0.0290885 | 0.0379488 | 0.443368 |
|  | rs1571878 | 6 | 167540842 | T | C | 0.5532 | -0.1355 | 0.0227 | 2.23599E-09 | 35.63050523 |  | T | C | 0.565974 | 0.0254566 | 0.0270005 | 0.345773 |
|  | rs2476601 | 1 | 114377568 | G | A | 0.8515 | -0.4652 | 0.0323 | 5.48277E-47 | 207.4289992 |  | G | A | 0.901772 | 0.0255802 | 0.0454507 | 0.573563 |
|  | rs34434863 | 6 | 32559673 | G | T | 0.3395 | 0.9684 | 0.0315 | 1E-200 | 945.1126118 |  | G | T | 0.290075 | -0.033168 | 0.0305722 | 0.277963 |
|  | rs34536443 | 19 | 10463118 | C | G | 0.03042 | -0.4029 | 0.0681 | 3.25297E-09 | 35.00220593 |  | C | G | 0.0401 | 0.0136386 | 0.0690863 | 0.843504 |
|  | rs34892989 | 6 | 33799390 | A | G | 0.2073 | -0.2116 | 0.0292 | 4.57826E-13 | 52.51226227 |  | A | G | 0.2598 | 0.0133247 | 0.0310469 | 0.667792 |
|  | rs4073285 | 1 | 2539796 | T | C | 0.3623 | -0.1152 | 0.0233 | 7.48893E-07 | 24.44489751 |  | T | C | 0.329333 | 0.0495257 | 0.0287761 | 0.0852374 |
|  | rs62395272 | 6 | 31394424 | T | C | 0.1068 | 0.4882 | 0.0374 | 6.68652E-39 | 170.3913133 |  | T | C | 0.090347 | 0.0152696 | 0.0473968 | 0.747328 |
|  | rs6568622 | 6 | 110298307 | C | A | 0.7893 | -0.1293 | 0.0274 | 2.31201E-06 | 22.26850331 |  | C | A | 0.754935 | 0.00145658 | 0.0312886 | 0.962869 |
|  | rs7137828 | 12 | 111932800 | T | C | 0.5857 | -0.1138 | 0.0227 | 5.32194E-07 | 25.13204996 |  | T | C | 0.511918 | -0.00769098 | 0.0265336 | 0.771925 |
|  | rs72976823 | 6 | 138019512 | A | G | 0.3294 | -0.1242 | 0.0239 | 1.92899E-07 | 27.00489508 |  | A | G | 0.231156 | 0.03451 | 0.032247 | 0.284541 |
|  | rs771289 | 2 | 7066555 | T | G | 0.2976 | -0.1125 | 0.0246 | 4.75094E-06 | 20.9136548 |  | T | G | 0.231867 | 0.0425367 | 0.0323969 | 0.189188 |
|  | rs7731626 | 5 | 55444683 | A | G | 0.2799 | -0.1397 | 0.0251 | 2.46201E-08 | 30.97707975 |  | A | G | 0.378205 | 0.0390756 | 0.0281201 | 0.164651 |
|  | rs78222069 | 4 | 123213720 | T | C | 0.01602 | 0.4331 | 0.0923 | 2.70601E-06 | 22.01750329 |  | T | C | 0.022235 | -0.100382 | 0.0916431 | 0.273362 |
|  | rs78795325 | 10 | 71598195 | G | A | 0.04199 | 0.2595 | 0.0566 | 4.49097E-06 | 21.02020594 |  | G | A | 0.056561 | 0.0467173 | 0.058004 | 0.42058 |
|  | rs7898906 | 10 | 63815937 | C | T | 0.5048 | -0.1053 | 0.0226 | 3.13603E-06 | 21.70875757 |  | C | T | 0.644916 | -0.0109106 | 0.0288236 | 0.705037 |
|  | rs8179673 | 2 | 191969341 | T | C | 0.7682 | -0.1361 | 0.0266 | 3.19897E-07 | 26.17870033 |  | T | C | 0.772159 | -0.0101953 | 0.0322516 | 0.751914 |
|  | rs9261601 | 6 | 30216146 | G | A | 0.4379 | 0.23 | 0.0243 | 2.48714E-21 | 89.58560468 |  | G | A | 0.476035 | 0.00261523 | 0.0270205 | 0.922896 |
|  | rs9272320 | 6 | 32604124 | A | G | 0.4287 | 0.474 | 0.0246 | 6.23735E-83 | 371.262918 |  | A | G | 0.595644 | -0.0134456 | 0.0274713 | 0.62453 |
|  | rs9277516 | 6 | 33054280 | C | T | 0.2548 | -0.278 | 0.0275 | 4.15528E-24 | 102.1925671 |  | C | T | 0.316009 | 0.000847152 | 0.0292501 | 0.976895 |
|  | rs932036 | 4 | 26090862 | T | A | 0.283 | 0.1338 | 0.0248 | 7.04904E-08 | 29.10743724 |  | T | A | 0.30174 | 0.0327055 | 0.0294166 | 0.266222 |
| Seronegative RA | rs10947233 | 6 | 32124424 | T | G | 0.0471 | 0.5658 | 0.082 | 5.2626E-12 | 47.60945517 | CagA | T | G | 0.061385 | 0.00848949 | 0.0911896 | 0.925826 |
|  | rs145575084 | 6 | 31365089 | C | G | 0.07452 | 0.873 | 0.0682 | 1.48696E-37 | 163.8531399 |  | C | G | 0.045645 | -0.0389972 | 0.104929 | 0.710151 |
|  | rs6679677 | 1 | 114303808 | A | C | 0.146 | 0.3121 | 0.0473 | 4.32116E-11 | 43.53717905 |  | A | C | 0.102308 | 0.0864434 | 0.0722623 | 0.231601 |
|  | rs9274507 | 6 | 32634152 | A | G | 0.7377 | 0.3863 | 0.0406 | 1.96879E-21 | 90.52997058 |  | A | G | 0.77152 | 0.120719 | 0.0517685 | 0.0197061 |
|  | rs9296004 | 6 | 31933977 | C | A | 0.09656 | 0.3251 | 0.0566 | 9.39399E-09 | 32.99104763 |  | C | A | 0.087368 | -0.0206756 | 0.0782407 | 0.791582 |
|  | rs10168266 | 2 | 191935804 | T | C | 0.2024 | 0.2074 | 0.0415 | 5.80898E-07 | 24.97562361 | OMP | T | C | 0.184998 | -0.00488184 | 0.0356181 | 0.890983 |
|  | rs142678586 | 3 | 6804479 | A | G | 0.00188 | 2.0712 | 0.4473 | 3.64998E-06 | 21.44081594 |  | A | G | 0.015941 | 0.15573 | 0.108316 | 0.15051 |
|  | rs145575084 | 6 | 31365089 | C | G | 0.07452 | 0.873 | 0.0682 | 1.48696E-37 | 163.8531399 |  | C | G | 0.041586 | 0.0348258 | 0.0682456 | 0.609842 |
|  | rs17120968 | 11 | 117493839 | T | C | 0.01964 | 0.5819 | 0.1223 | 1.93798E-06 | 22.63804039 |  | T | C | 0.022062 | -0.0706285 | 0.0943285 | 0.454009 |
|  | rs2476601 | 1 | 114377568 | G | A | 0.8526 | -0.3212 | 0.0472 | 9.96093E-12 | 46.30864846 |  | G | A | 0.901282 | 0.0331381 | 0.0470769 | 0.481486 |
|  | rs304729 | 19 | 44084403 | A | G | 0.1779 | 0.1996 | 0.043 | 0.000003457 | 21.54662201 |  | A | G | 0.190828 | 0.00093568 | 0.0348273 | 0.978566 |
|  | rs34057012 | 16 | 89743083 | A | G | 0.4519 | -0.1572 | 0.0336 | 2.84702E-06 | 21.88878012 |  | A | G | 0.356918 | 0.0616072 | 0.0291575 | 0.0346083 |
|  | rs4660553 | 1 | 42134604 | A | G | 0.07236 | 0.2971 | 0.0649 | 0.000004685 | 20.95612306 |  | A | G | 0.042607 | -0.0149756 | 0.0685529 | 0.827076 |
|  | rs4902632 | 14 | 69149428 | T | A | 0.7555 | -0.1797 | 0.0389 | 3.77798E-06 | 21.33988043 |  | T | A | 0.8223 | 0.0372548 | 0.0367236 | 0.310361 |
|  | rs5761256 | 22 | 26218164 | G | A | 0.2963 | 0.1753 | 0.0364 | 1.45101E-06 | 23.19295551 |  | G | A | 0.276331 | 0.0270194 | 0.0310982 | 0.384935 |
|  | rs72933309 | 6 | 109194634 | T | C | 0.04742 | -0.3687 | 0.0807 | 0.000004889 | 20.87345 |  | T | C | 0.012666 | 0.27772 | 0.122277 | 0.0231324 |
|  | rs74483355 | 16 | 30836430 | T | C | 0.06392 | 0.3235 | 0.068 | 1.98098E-06 | 22.63214801 |  | T | C | 0.109685 | 0.013524 | 0.044779 | 0.762639 |
|  | rs75132258 | 10 | 126812002 | T | C | 0.0497 | 0.3641 | 0.0774 | 2.54701E-06 | 22.12862691 |  | T | C | 0.060683 | -0.00789396 | 0.0581554 | 0.892028 |
|  | rs75351188 | 12 | 117005064 | T | G | 0.05263 | -0.3515 | 0.076 | 3.70502E-06 | 21.39038022 |  | T | G | 0.056407 | 0.0208715 | 0.0601482 | 0.72859 |
|  | rs75549101 | 4 | 11910875 | C | G | 0.01096 | 0.7694 | 0.1662 | 3.66201E-06 | 21.43074926 |  | C | G | 0.027259 | 0.156035 | 0.085307 | 0.0673845 |
|  | rs7731626 | 5 | 55444683 | A | G | 0.2802 | -0.1743 | 0.0369 | 2.35201E-06 | 22.31192657 |  | A | G | 0.38049 | 0.024314 | 0.0290486 | 0.402586 |
|  | rs78502259 | 5 | 138752403 | A | G | 0.02547 | 0.4986 | 0.109 | 4.79104E-06 | 20.92409015 |  | A | G | 0.036922 | -0.136892 | 0.0737095 | 0.0632863 |
|  | rs78525147 | 3 | 115614340 | A | C | 0.01979 | 0.5994 | 0.1268 | 2.25699E-06 | 22.34549606 |  | A | C | 0.028177 | 0.0133081 | 0.0839794 | 0.874088 |
|  | rs9261599 | 6 | 30214146 | T | G | 0.4549 | 0.1941 | 0.0357 | 5.25303E-08 | 29.56035659 |  | T | G | 0.488528 | 0.0131834 | 0.0282279 | 0.640475 |
|  | rs9296004 | 6 | 31933977 | C | A | 0.09656 | 0.3251 | 0.0566 | 9.39399E-09 | 32.99104763 |  | C | A | 0.086973 | -0.0474527 | 0.0488338 | 0.331191 |
|  | rs10168266 | 2 | 191935804 | T | C | 0.2024 | 0.2074 | 0.0415 | 5.80898E-07 | 24.97562361 | IgG | T | C | 0.190212 | -0.00765081 | 0.00859619 | 0.373453 |
|  | rs142678586 | 3 | 6804479 | A | G | 0.00188 | 2.0712 | 0.4473 | 3.64998E-06 | 21.44081594 |  | A | G | 0.018094 | -0.00683795 | 0.0251088 | 0.785366 |
|  | rs145575084 | 6 | 31365089 | C | G | 0.07452 | 0.873 | 0.0682 | 1.48696E-37 | 163.8531399 |  | C | G | 0.044778 | -0.00743489 | 0.0162267 | 0.646818 |
|  | rs17120968 | 11 | 117493839 | T | C | 0.01964 | 0.5819 | 0.1223 | 1.93798E-06 | 22.63804039 |  | T | C | 0.020785 | -0.0064483 | 0.0238796 | 0.787135 |
|  | rs2476601 | 1 | 114377568 | G | A | 0.8526 | -0.3212 | 0.0472 | 9.96093E-12 | 46.30864846 |  | G | A | 0.902003 | 0.0023267 | 0.0113749 | 0.837927 |
|  | rs304729 | 19 | 44084403 | A | G | 0.1779 | 0.1996 | 0.043 | 0.000003457 | 21.54662201 |  | A | G | 0.18566 | -0.00369948 | 0.00868782 | 0.670236 |
|  | rs34057012 | 16 | 89743083 | A | G | 0.4519 | -0.1572 | 0.0336 | 2.84702E-06 | 21.88878012 |  | A | G | 0.361237 | -0.015776 | 0.00712066 | 0.0267239 |
|  | rs4660553 | 1 | 42134604 | A | G | 0.07236 | 0.2971 | 0.0649 | 0.000004685 | 20.95612306 |  | A | G | 0.040268 | 0.0266394 | 0.017203 | 0.121493 |
|  | rs4902632 | 14 | 69149428 | T | A | 0.7555 | -0.1797 | 0.0389 | 3.77798E-06 | 21.33988043 |  | T | A | 0.832968 | -0.00831518 | 0.00925373 | 0.368878 |
|  | rs5761256 | 22 | 26218164 | G | A | 0.2963 | 0.1753 | 0.0364 | 1.45101E-06 | 23.19295551 |  | G | A | 0.283343 | 0.00627253 | 0.00750969 | 0.403572 |
|  | rs72933309 | 6 | 109194634 | T | C | 0.04742 | -0.3687 | 0.0807 | 0.000004889 | 20.87345 |  | T | C | 0.014341 | -0.0599683 | 0.0284353 | 0.0349502 |
|  | rs74483355 | 16 | 30836430 | T | C | 0.06392 | 0.3235 | 0.068 | 1.98098E-06 | 22.63214801 |  | T | C | 0.113741 | -0.00594846 | 0.0106931 | 0.578012 |
|  | rs75132258 | 10 | 126812002 | T | C | 0.0497 | 0.3641 | 0.0774 | 2.54701E-06 | 22.12862691 |  | T | C | 0.060093 | -0.00281702 | 0.0142989 | 0.84382 |
|  | rs75351188 | 12 | 117005064 | T | G | 0.05263 | -0.3515 | 0.076 | 3.70502E-06 | 21.39038022 |  | T | G | 0.054187 | -0.0202847 | 0.0149125 | 0.173752 |
|  | rs75549101 | 4 | 11910875 | C | G | 0.01096 | 0.7694 | 0.1662 | 3.66201E-06 | 21.43074926 |  | C | G | 0.032014 | -0.0326568 | 0.0192555 | 0.0898918 |
|  | rs7731626 | 5 | 55444683 | A | G | 0.2802 | -0.1743 | 0.0369 | 2.35201E-06 | 22.31192657 |  | A | G | 0.380481 | -0.00581869 | 0.00702778 | 0.407696 |
|  | rs78502259 | 5 | 138752403 | A | G | 0.02547 | 0.4986 | 0.109 | 4.79104E-06 | 20.92409015 |  | A | G | 0.033944 | 0.00310434 | 0.0188995 | 0.86953 |
|  | rs78525147 | 3 | 115614340 | A | C | 0.01979 | 0.5994 | 0.1268 | 2.25699E-06 | 22.34549606 |  | A | C | 0.026274 | 0.0140887 | 0.0209925 | 0.502137 |
|  | rs9261599 | 6 | 30214146 | T | G | 0.4549 | 0.1941 | 0.0357 | 5.25303E-08 | 29.56035659 |  | T | G | 0.494156 | -0.0038057 | 0.00683277 | 0.577543 |
|  | rs9271376 | 6 | 32587113 | A | G | 0.7288 | 0.3985 | 0.0406 | 8.85727E-23 | 96.33844108 |  | A | G | 0.746805 | 0.00798444 | 0.00787015 | 0.310334 |
|  | rs9296004 | 6 | 31933977 | C | A | 0.09656 | 0.3251 | 0.0566 | 9.39399E-09 | 32.99104763 |  | C | A | 0.082835 | 0.0330398 | 0.0122725 | 0.00709839 |
|  | rs10168266 | 2 | 191935804 | T | C | 0.2024 | 0.2074 | 0.0415 | 5.80898E-07 | 24.97562361 | UREA | T | C | 0.190563 | 0.00558487 | 0.0379025 | 0.882857 |
|  | rs142678586 | 3 | 6804479 | A | G | 0.00188 | 2.0712 | 0.4473 | 3.64998E-06 | 21.44081594 |  | A | G | 0.016561 | -0.00820411 | 0.115145 | 0.943199 |
|  | rs145575084 | 6 | 31365089 | C | G | 0.07452 | 0.873 | 0.0682 | 1.48696E-37 | 163.8531399 |  | C | G | 0.04333 | -0.0382852 | 0.0726212 | 0.598062 |
|  | rs17120968 | 11 | 117493839 | T | C | 0.01964 | 0.5819 | 0.1223 | 1.93798E-06 | 22.63804039 |  | T | C | 0.021165 | 0.0283096 | 0.102797 | 0.783015 |
|  | rs2476601 | 1 | 114377568 | G | A | 0.8526 | -0.3212 | 0.0472 | 9.96093E-12 | 46.30864846 |  | G | A | 0.908349 | 0.00682744 | 0.0512803 | 0.894083 |
|  | rs304729 | 19 | 44084403 | A | G | 0.1779 | 0.1996 | 0.043 | 0.000003457 | 21.54662201 |  | A | G | 0.186706 | 0.0261774 | 0.0377348 | 0.487858 |
|  | rs34057012 | 16 | 89743083 | A | G | 0.4519 | -0.1572 | 0.0336 | 2.84702E-06 | 21.88878012 |  | A | G | 0.363029 | -0.0744332 | 0.0312004 | 0.017049 |
|  | rs4660553 | 1 | 42134604 | A | G | 0.07236 | 0.2971 | 0.0649 | 0.000004685 | 20.95612306 |  | A | G | 0.044485 | -0.0862974 | 0.0725798 | 0.23444 |
|  | rs4902632 | 14 | 69149428 | T | A | 0.7555 | -0.1797 | 0.0389 | 3.77798E-06 | 21.33988043 |  | T | A | 0.831957 | -0.0466419 | 0.0402572 | 0.24662 |
|  | rs5761256 | 22 | 26218164 | G | A | 0.2963 | 0.1753 | 0.0364 | 1.45101E-06 | 23.19295551 |  | G | A | 0.29696 | -0.0502214 | 0.0324737 | 0.121978 |
|  | rs72933309 | 6 | 109194634 | T | C | 0.04742 | -0.3687 | 0.0807 | 0.000004889 | 20.87345 |  | T | C | 0.012523 | 0.193164 | 0.129165 | 0.134788 |
|  | rs74483355 | 16 | 30836430 | T | C | 0.06392 | 0.3235 | 0.068 | 1.98098E-06 | 22.63214801 |  | T | C | 0.113306 | 0.0147604 | 0.0470918 | 0.753947 |
|  | rs75132258 | 10 | 126812002 | T | C | 0.0497 | 0.3641 | 0.0774 | 2.54701E-06 | 22.12862691 |  | T | C | 0.061985 | 0.00154521 | 0.0606742 | 0.979682 |
|  | rs75351188 | 12 | 117005064 | T | G | 0.05263 | -0.3515 | 0.076 | 3.70502E-06 | 21.39038022 |  | T | G | 0.052294 | -0.0134133 | 0.0670337 | 0.841404 |
|  | rs75549101 | 4 | 11910875 | C | G | 0.01096 | 0.7694 | 0.1662 | 3.66201E-06 | 21.43074926 |  | C | G | 0.032742 | -0.139983 | 0.0832812 | 0.0927919 |
|  | rs7731626 | 5 | 55444683 | A | G | 0.2802 | -0.1743 | 0.0369 | 2.35201E-06 | 22.31192657 |  | A | G | 0.385662 | 0.000959303 | 0.0307345 | 0.9751 |
|  | rs78502259 | 5 | 138752403 | A | G | 0.02547 | 0.4986 | 0.109 | 4.79104E-06 | 20.92409015 |  | A | G | 0.032668 | -0.117378 | 0.0838987 | 0.161801 |
|  | rs78525147 | 3 | 115614340 | A | C | 0.01979 | 0.5994 | 0.1268 | 2.25699E-06 | 22.34549606 |  | A | C | 0.025862 | 0.161497 | 0.0925872 | 0.0811129 |
|  | rs9261599 | 6 | 30214146 | T | G | 0.4549 | 0.1941 | 0.0357 | 5.25303E-08 | 29.56035659 |  | T | G | 0.492582 | 0.0495466 | 0.0298105 | 0.0965028 |
|  | rs9271376 | 6 | 32587113 | A | G | 0.7288 | 0.3985 | 0.0406 | 8.85727E-23 | 96.33844108 |  | A | G | 0.75503 | 0.0430764 | 0.0343282 | 0.209536 |
|  | rs9296004 | 6 | 31933977 | C | A | 0.09656 | 0.3251 | 0.0566 | 9.39399E-09 | 32.99104763 |  | C | A | 0.093352 | -0.0363759 | 0.0506212 | 0.472393 |
|  | rs10168266 | 2 | 191935804 | T | C | 0.2024 | 0.2074 | 0.0415 | 5.80898E-07 | 24.97562361 | VacA | T | C | 0.189163 | -0.0187655 | 0.045517 | 0.680138 |
|  | rs142678586 | 3 | 6804479 | A | G | 0.00188 | 2.0712 | 0.4473 | 3.64998E-06 | 21.44081594 |  | A | G | 0.022388 | -0.0159612 | 0.119039 | 0.893337 |
|  | rs145575084 | 6 | 31365089 | C | G | 0.07452 | 0.873 | 0.0682 | 1.48696E-37 | 163.8531399 |  | C | G | 0.041558 | 0.0946682 | 0.0882895 | 0.283609 |
|  | rs17120968 | 11 | 117493839 | T | C | 0.01964 | 0.5819 | 0.1223 | 1.93798E-06 | 22.63804039 |  | T | C | 0.021214 | -0.0573644 | 0.124333 | 0.644528 |
|  | rs2476601 | 1 | 114377568 | G | A | 0.8526 | -0.3212 | 0.0472 | 9.96093E-12 | 46.30864846 |  | G | A | 0.904283 | -0.0589402 | 0.0609924 | 0.333868 |
|  | rs304729 | 19 | 44084403 | A | G | 0.1779 | 0.1996 | 0.043 | 0.000003457 | 21.54662201 |  | A | G | 0.192732 | -0.00774367 | 0.0439128 | 0.860025 |
|  | rs34057012 | 16 | 89743083 | A | G | 0.4519 | -0.1572 | 0.0336 | 2.84702E-06 | 21.88878012 |  | A | G | 0.358689 | 0.0530549 | 0.0368638 | 0.15009 |
|  | rs4660553 | 1 | 42134604 | A | G | 0.07236 | 0.2971 | 0.0649 | 0.000004685 | 20.95612306 |  | A | G | 0.045809 | 0.0330268 | 0.0834776 | 0.692373 |
|  | rs4902632 | 14 | 69149428 | T | A | 0.7555 | -0.1797 | 0.0389 | 3.77798E-06 | 21.33988043 |  | T | A | 0.822702 | -0.00653273 | 0.0467834 | 0.888946 |
|  | rs5761256 | 22 | 26218164 | G | A | 0.2963 | 0.1753 | 0.0364 | 1.45101E-06 | 23.19295551 |  | G | A | 0.291369 | 0.00950821 | 0.0388762 | 0.806784 |
|  | rs72933309 | 6 | 109194634 | T | C | 0.04742 | -0.3687 | 0.0807 | 0.000004889 | 20.87345 |  | T | C | 0.014629 | -0.0336991 | 0.145136 | 0.81639 |
|  | rs74483355 | 16 | 30836430 | T | C | 0.06392 | 0.3235 | 0.068 | 1.98098E-06 | 22.63214801 |  | T | C | 0.104938 | 0.098446 | 0.0578299 | 0.0886911 |
|  | rs75132258 | 10 | 126812002 | T | C | 0.0497 | 0.3641 | 0.0774 | 2.54701E-06 | 22.12862691 |  | T | C | 0.059305 | -0.045942 | 0.0753326 | 0.541957 |
|  | rs75351188 | 12 | 117005064 | T | G | 0.05263 | -0.3515 | 0.076 | 3.70502E-06 | 21.39038022 |  | T | G | 0.047368 | -0.0551945 | 0.0846192 | 0.514228 |
|  | rs75549101 | 4 | 11910875 | C | G | 0.01096 | 0.7694 | 0.1662 | 3.66201E-06 | 21.43074926 |  | C | G | 0.032878 | 0.0736926 | 0.0999167 | 0.460794 |
|  | rs7731626 | 5 | 55444683 | A | G | 0.2802 | -0.1743 | 0.0369 | 2.35201E-06 | 22.31192657 |  | A | G | 0.386113 | 0.0344631 | 0.037199 | 0.35421 |
|  | rs78502259 | 5 | 138752403 | A | G | 0.02547 | 0.4986 | 0.109 | 4.79104E-06 | 20.92409015 |  | A | G | 0.033744 | -0.0717354 | 0.0995964 | 0.471364 |
|  | rs78525147 | 3 | 115614340 | A | C | 0.01979 | 0.5994 | 0.1268 | 2.25699E-06 | 22.34549606 |  | A | C | 0.028877 | -0.071818 | 0.107108 | 0.502525 |
|  | rs9261599 | 6 | 30214146 | T | G | 0.4549 | 0.1941 | 0.0357 | 5.25303E-08 | 29.56035659 |  | T | G | 0.494386 | 0.00908402 | 0.0358481 | 0.799957 |
|  | rs9271376 | 6 | 32587113 | A | G | 0.7288 | 0.3985 | 0.0406 | 8.85727E-23 | 96.33844108 |  | A | G | 0.740081 | 0.0179042 | 0.0400069 | 0.654494 |
|  | rs9296004 | 6 | 31933977 | C | A | 0.09656 | 0.3251 | 0.0566 | 9.39399E-09 | 32.99104763 |  | C | A | 0.0842 | 0.0799213 | 0.0647387 | 0.217009 |
|  | rs10168266 | 2 | 191935804 | T | C | 0.2024 | 0.2074 | 0.0448579 | 0.55541 | 24.97562361 | Catalase | T | C | 0.186763 | -0.0264515 | 0.0415 | 5.80898E-07 |
|  | rs142678586 | 3 | 6804479 | A | G | 0.00188 | 2.0712 | 0.156331 | 0.543999 | 21.44081594 |  | A | G | 0.013106 | -0.0948583 | 0.4473 | 3.64998E-06 |
|  | rs145575084 | 6 | 31365089 | C | G | 0.07452 | 0.873 | 0.0838843 | 0.295239 | 163.8531399 |  | C | G | 0.04751 | 0.0878015 | 0.0682 | 1.48696E-37 |
|  | rs17120968 | 11 | 117493839 | T | C | 0.01964 | 0.5819 | 0.12107 | 0.889196 | 22.63804039 |  | T | C | 0.022354 | 0.0168675 | 0.1223 | 1.93798E-06 |
|  | rs2476601 | 1 | 114377568 | G | A | 0.8526 | -0.3212 | 0.0612959 | 0.510143 | 46.30864846 |  | G | A | 0.90367 | 0.0403704 | 0.0472 | 9.96093E-12 |
|  | rs304729 | 19 | 44084403 | A | G | 0.1779 | 0.1996 | 0.0451544 | 0.0259406 | 21.54662201 |  | A | G | 0.179882 | 0.100563 | 0.043 | 0.000003457 |
|  | rs34057012 | 16 | 89743083 | A | G | 0.4519 | -0.1572 | 0.0376652 | 0.254213 | 21.88878012 |  | A | G | 0.350232 | 0.042945 | 0.0336 | 2.84702E-06 |
|  | rs4660553 | 1 | 42134604 | A | G | 0.07236 | 0.2971 | 0.086898 | 0.90139 | 20.95612306 |  | A | G | 0.042267 | 0.0107672 | 0.0649 | 0.000004685 |
|  | rs4902632 | 14 | 69149428 | T | A | 0.7555 | -0.1797 | 0.0486995 | 0.604103 | 21.33988043 |  | T | A | 0.835522 | 0.0252511 | 0.0389 | 3.77798E-06 |
|  | rs5761256 | 22 | 26218164 | G | A | 0.2963 | 0.1753 | 0.0387934 | 0.368026 | 23.19295551 |  | G | A | 0.288663 | -0.0349209 | 0.0364 | 1.45101E-06 |
|  | rs72933309 | 6 | 109194634 | T | C | 0.04742 | -0.3687 | 0.156126 | 0.477522 | 20.87345 |  | T | C | 0.012492 | 0.110895 | 0.0807 | 0.000004889 |
|  | rs74483355 | 16 | 30836430 | T | C | 0.06392 | 0.3235 | 0.0559912 | 0.934494 | 22.63214801 |  | T | C | 0.114679 | -0.00460201 | 0.068 | 1.98098E-06 |
|  | rs75132258 | 10 | 126812002 | T | C | 0.0497 | 0.3641 | 0.0749767 | 0.265186 | 22.12862691 |  | T | C | 0.057096 | -0.0835402 | 0.0774 | 2.54701E-06 |
|  | rs75351188 | 12 | 117005064 | T | G | 0.05263 | -0.3515 | 0.0794912 | 0.373037 | 21.39038022 |  | T | G | 0.052194 | -0.0708107 | 0.076 | 3.70502E-06 |
|  | rs75549101 | 4 | 11910875 | C | G | 0.01096 | 0.7694 | 0.107732 | 0.615608 | 21.43074926 |  | C | G | 0.028581 | -0.0540909 | 0.1662 | 3.66201E-06 |
|  | rs7731626 | 5 | 55444683 | A | G | 0.2802 | -0.1743 | 0.0374043 | 0.499596 | 22.31192657 |  | A | G | 0.374836 | 0.0252526 | 0.0369 | 2.35201E-06 |
|  | rs78502259 | 5 | 138752403 | A | G | 0.02547 | 0.4986 | 0.0994075 | 0.416185 | 20.92409015 |  | A | G | 0.033093 | 0.080824 | 0.109 | 4.79104E-06 |
|  | rs78525147 | 3 | 115614340 | A | C | 0.01979 | 0.5994 | 0.104269 | 0.718704 | 22.34549606 |  | A | C | 0.029817 | -0.0375568 | 0.1268 | 2.25699E-06 |
|  | rs9261599 | 6 | 30214146 | T | G | 0.4549 | 0.1941 | 0.0348786 | 0.622726 | 29.56035659 |  | T | G | 0.484656 | 0.01716 | 0.0357 | 5.25303E-08 |
|  | rs9271376 | 6 | 32587113 | A | G | 0.7288 | 0.3985 | 0.0434217 | 0.00576514 | 96.33844108 |  | A | G | 0.781886 | 0.119881 | 0.0406 | 8.85727E-23 |
|  | rs9296004 | 6 | 31933977 | C | A | 0.09656 | 0.3251 | 0.0606064 | 0.83284 | 32.99104763 |  | C | A | 0.091327 | 0.0127917 | 0.0566 | 9.39399E-09 |
|  | rs10168266 | 2 | 191935804 | T | C | 0.2024 | 0.2074 | 0.0415 | 5.80898E-07 | 24.97562361 | GroEL | T | C | 0.191365 | 0.0024633 | 0.0339225 | 0.942112 |
|  | rs142678586 | 3 | 6804479 | A | G | 0.00188 | 2.0712 | 0.4473 | 3.64998E-06 | 21.44081594 |  | A | G | 0.019057 | -0.104027 | 0.0972033 | 0.28453 |
|  | rs145575084 | 6 | 31365089 | C | G | 0.07452 | 0.873 | 0.0682 | 1.48696E-37 | 163.8531399 |  | C | G | 0.044306 | -0.0693691 | 0.0652936 | 0.288046 |
|  | rs17120968 | 11 | 117493839 | T | C | 0.01964 | 0.5819 | 0.1223 | 1.93798E-06 | 22.63804039 |  | T | C | 0.020509 | 0.0506145 | 0.0960612 | 0.598264 |
|  | rs2476601 | 1 | 114377568 | G | A | 0.8526 | -0.3212 | 0.0472 | 9.96093E-12 | 46.30864846 |  | G | A | 0.901772 | 0.0255802 | 0.0454507 | 0.573563 |
|  | rs304729 | 19 | 44084403 | A | G | 0.1779 | 0.1996 | 0.043 | 0.000003457 | 21.54662201 |  | A | G | 0.187925 | 0.0688468 | 0.0342304 | 0.0442966 |
|  | rs34057012 | 16 | 89743083 | A | G | 0.4519 | -0.1572 | 0.0336 | 2.84702E-06 | 21.88878012 |  | A | G | 0.356462 | 0.0261201 | 0.0286933 | 0.362653 |
|  | rs4660553 | 1 | 42134604 | A | G | 0.07236 | 0.2971 | 0.0649 | 0.000004685 | 20.95612306 |  | A | G | 0.040566 | 0.0923957 | 0.0675328 | 0.171262 |
|  | rs4902632 | 14 | 69149428 | T | A | 0.7555 | -0.1797 | 0.0389 | 3.77798E-06 | 21.33988043 |  | T | A | 0.829137 | -0.0231539 | 0.0362619 | 0.523136 |
|  | rs5761256 | 22 | 26218164 | G | A | 0.2963 | 0.1753 | 0.0364 | 1.45101E-06 | 23.19295551 |  | G | A | 0.285068 | 0.00805814 | 0.0292908 | 0.783233 |
|  | rs72933309 | 6 | 109194634 | T | C | 0.04742 | -0.3687 | 0.0807 | 0.000004889 | 20.87345 |  | T | C | 0.012283 | 0.0840224 | 0.119076 | 0.480425 |
|  | rs74483355 | 16 | 30836430 | T | C | 0.06392 | 0.3235 | 0.068 | 1.98098E-06 | 22.63214801 |  | T | C | 0.11656 | -0.0563505 | 0.0429565 | 0.189586 |
|  | rs75132258 | 10 | 126812002 | T | C | 0.0497 | 0.3641 | 0.0774 | 2.54701E-06 | 22.12862691 |  | T | C | 0.062357 | 0.0212864 | 0.0556058 | 0.701862 |
|  | rs75351188 | 12 | 117005064 | T | G | 0.05263 | -0.3515 | 0.076 | 3.70502E-06 | 21.39038022 |  | T | G | 0.051829 | -0.0967657 | 0.0604194 | 0.109252 |
|  | rs75549101 | 4 | 11910875 | C | G | 0.01096 | 0.7694 | 0.1662 | 3.66201E-06 | 21.43074926 |  | C | G | 0.028345 | -0.0059538 | 0.0816102 | 0.941843 |
|  | rs7731626 | 5 | 55444683 | A | G | 0.2802 | -0.1743 | 0.0369 | 2.35201E-06 | 22.31192657 |  | A | G | 0.378205 | 0.0390756 | 0.0281201 | 0.164651 |
|  | rs78502259 | 5 | 138752403 | A | G | 0.02547 | 0.4986 | 0.109 | 4.79104E-06 | 20.92409015 |  | A | G | 0.032428 | -0.0216078 | 0.0766088 | 0.777902 |
|  | rs78525147 | 3 | 115614340 | A | C | 0.01979 | 0.5994 | 0.1268 | 2.25699E-06 | 22.34549606 |  | A | C | 0.027338 | 0.112233 | 0.0828983 | 0.175781 |
|  | rs9261599 | 6 | 30214146 | T | G | 0.4549 | 0.1941 | 0.0357 | 5.25303E-08 | 29.56035659 |  | T | G | 0.495779 | -0.00438842 | 0.0270743 | 0.871237 |
|  | rs9271376 | 6 | 32587113 | A | G | 0.7288 | 0.3985 | 0.0406 | 8.85727E-23 | 96.33844108 |  | A | G | 0.749417 | 0.0340794 | 0.0307995 | 0.268513 |
|  | rs9296004 | 6 | 31933977 | C | A | 0.09656 | 0.3251 | 0.0566 | 9.39399E-09 | 32.99104763 |  | C | A | 0.090291 | -0.009779 | 0.046729 | 0.834237 |
| Allele effects on Exposure: the association summary statistics of the SNP on exposure. Allele effects on Outcome: the association summary statistics of the SNP on outcome. | | | | | | | | | | | | | | | | | |
